# Extracellular vesicles and their cargo molecules for peripheral nerve injuries and neuropathies: The composition, properties, and impact

**DOI:** 10.1016/j.mam.2026.101485

**Published:** 2026-06-10

**Authors:** Anam Anjum, Abel Lindley, Meaghan Harley-Troxell, Xiaofeng Jia

**Affiliations:** a Department of Neurosurgery, University of Maryland School of Medicine, Baltimore, MD, 21201, USA; b Department of Orthopedics, University of Maryland School of Medicine, Baltimore, MD, 21201, USA; c Department of Neurobiology, University of Maryland School of Medicine, Baltimore, MD, 21201, USA; d Fischell Department of Bioengineering, University of Maryland, College Park, MD, 20742, USA

**Keywords:** Peripheral nerve injury (PNI), Peripheral neuropathies, Stem cells, Extracellular vesicles (EV), Exosomes, Nanocarriers, Nerve regeneration, Signaling pathways

## Abstract

Peripheral nerve repair remains a major clinical challenge due to limited functional recovery and the lack of effective therapeutic options. Extracellular vesicles (EVs) have emerged as potential mediators of nerve regeneration due to their roles in cell-to-cell communication and the delivery of diverse bioactive molecules. The regenerative potential of EVs largely depends on the type of cell they originate from, which determines their molecular cargo and therapeutic potential. This review provides a comprehensive and focused analysis on the source-specific composition of EVs, and how different EVs distinctly influence neuroprotective, immunomodulatory, and neuroregenerative properties with direct relevance to peripheral nerve injury (PNI). We also highlight current evidence on how EVs modulate nerve repair pathways and discuss the implications of their source-specific content in optimizing EV-based therapies alongside recent technological advances for PNI. By highlighting key differences in regenerative-associated signaling, this review provides insight into optimizing EV-based therapies for PNI and outlines prospects for clinical application.

## 1. Introduction

Peripheral nerve injury (PNI) is a debilitating outcome of traumatic events such as motor vehicle accidents, workplace injuries, and sports-related incidents. PNI often results in demyelination, such as Wallerian degeneration, characterized by the breakdown of axonal and myelin structures. In response, Schwann cells (SCs), the primary glial cells of the peripheral nervous system (PNS), can dedifferentiate into a non-myelinating repair phenotype that actively breaks down myelin. While this transition is necessary for eventual healing, the accumulation of axonal debris, if not efficiently cleared, can significantly hinder regeneration (Nocera and Jacob, 2020). Current therapeutic strategies, including surgical nerve repair, autografting, and pharmacological interventions, remain limited by suboptimal functional recovery and considerable donor site morbidity, stemming largely from the inability to fully restore the complex microenvironment needed for effective nerve regeneration.

To overcome these limitations, cell-free therapies have emerged as potential adjuncts to traditional cell therapies. Among these are extracellular vesicles (EVs), which have garnered recent attention as a versatile, cell-free therapeutic modality. These membrane-bound particles, secreted by nearly all cell types, can be found in various biological fluids, including blood, urine, and bile. According to the Minimal Information for Studies of Extracellular Vesicles (MISEV) 2023 guidelines by the International Society for Extracellular Vesicles, EVs can be further classified into three main categories based on size and biogenesis: apoptotic bodies (500–5000 nm), microvesicles (100–1000 nm), and exosomes (30–150 nm), the smallest EV subtype (Consortium, 2024). Due to their small size, EVs in all three of these classifications have shown therapeutic promise that allows them to cross biological barriers while displaying surface molecules to modulate host cell signaling (Table 1) (Hulstaert et al., 2020). Notably, the term “exosome” should be applied cautiously, in line with MISEV recommendations, to avoid misclassification when origin or biogenesis is unclear. EVs are typically characterized through a combination of biophysical and molecular approaches, including nanoparticle tracking analysis, transmission electron microscopy, flow cytometry, and protein marker profiling (i.e. CD9, CD63, CD81) (Gonçalves et al., 2020; Xu et al., 2019a). In this review, we retain the term “exosome” when used in their cited publication to preserve the authors’ terminology, particularly when referring to precise therapeutic effects that may differ from broader EV classifications due to differences in size, isolation methods, vesicle heterogeneity, and cargo composition.

**Fig. 1.**
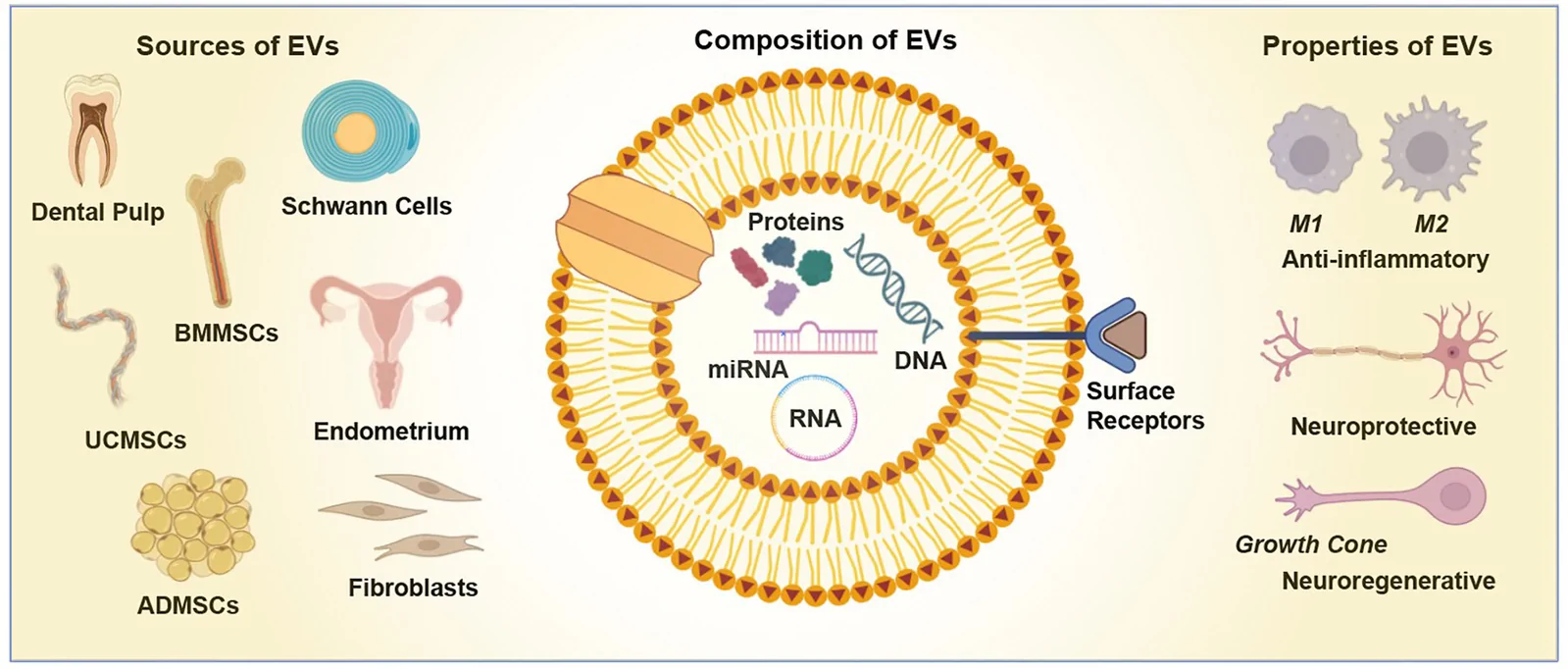
Sources of EVs isolated from SCs, bone marrow (BMMSCs), adipose tissue (ADMSCs), umbilical cord (UCMSCs), fibroblasts, skin precursor cells, and dental pulp. These EVs regulate nerve-related cellular functions and modulate key processes such as neuroinflammation, immune cell activity, neuroprotection, axonal regrowth, and remyelination.

Multiple benefits are provided by EVs: they deliver bioactive cargo to recipient cells, have a simpler structural design, are more consistent in their biological function, and have a greater ease of storage and customization (Yu et al., 2021). They can also be naturally derived from diverse sources, including SCs, mesenchymal stem cells (MSCs), dorsal root ganglions (DRGs), fibroblasts, immune cells, epithelial cells, osteocytes, and platelets (Fig. 1) (Cui et al., 2021; Fan et al., 2020). The origin of EVs is critical in determining their regenerative potential, as their molecular content closely reflects the phenotype and function of their parent cells. Consequentially, EVs serve as specialized mediators of intercellular communication via the delivery of lineage-specific molecular cargo that can precisely modulate recipient cell behavior to promote effective axonal regeneration (Saquel et al., 2022).

Although a growing number of studies have explored the application of EVs in nerve regeneration, existing reviews in this field are largely descriptive and tend to treat EVs as a relatively homogeneous therapeutic entity. This approach overlooks a critical aspect of EV biology: the functional specificity conferred by their source-specific cargo composition. To address these gaps, this review aims to explore a cargo-driven and source-comparative framework to evaluate the role of EVs in peripheral nerve repair and neuropathies. We will further discuss EVs’ unique composition and functional roles, with a focus on how microRNAs (miRNAs), proteins, and lipids regulate cellular processes essential for nerve regeneration. Finally, a critical comparison will be discussed to evaluate engineered and preconditioned EVs in both *in vitro* and *in vivo* models of PNI, integrating current preclinical evidence with emerging clinical insights to discuss the translational prospects and challenges of EV-based medicine. By linking molecular composition to functional outcomes and emphasizing source-dependent differences, this review moves beyond descriptive summaries and provides a more mechanistic and clinically relevant understanding of EV-mediated nerve repair.

### 1.1. EVs: impact on intercellular communication and transfer of biological information

EVs, including exosomes, are a heterogeneous population of membrane-bound particles, originating from the endosomal pathway. EV heterogeneity extends beyond size and biogenesis to include substantial variation in cargo composition, which is influenced by their parent cell type, physiological state, and environmental conditions. Differences in EV isolation methods, cellular sources, and culture conditions can also lead to significant variability in experimental outcomes.

Effective intercellular communication is essential for maintaining neural homeostasis, supporting development, and facilitating repair. Synaptic transmission and gap junctions are two highly regulated mechanisms through which this communication occurs. In the context of PNI, EV-mediated signaling is particularly critical for coordinating tissue regeneration and reinnervation through these mechanisms (Forero et al., 2024). Upon release into the extracellular space, EVs can interact with target cells through various mechanisms, such as surface receptor binding, endocytosis, or direct membrane fusion, as demonstrated in early experimental studies (Veerman et al., 2021). These interactions are governed by specific EV surface molecules: Tetraspanins (CD9, CD63, CD81) organize membrane microdomains that facilitate docking, while integrins (e.g., α6β1, αvβ3) mediate selective adhesion to extracellular matrix components such as laminin and fibronectin, enhancing targeting to neurons and SCs. Additionally, neural adhesion molecules including L1 Cell Adhesion Molecule (L1CAM) and Neural Cell Adhesion Molecule (NCAM) contribute to EV uptake by promoting their binding to axons and glial cells, supporting directed communication during nerve repair. As such, EVs play an essential role in promoting tissue remodeling and growth, while also aiding in differentiation through direct ligand-receptor interactions and gap junction coupling (Dagenais et al., 2021; Liu et al., 2024).

The functional role of EVs in PNI is determined by their interaction with SCs, neurons, and immune cells–key mediators of the nerve microenvironment. EVs enhance SC survival, migration, and remyelination while promoting neuronal axonal regeneration through growth signals and cytoskeletal remodeling. They also modulate immune responses by influencing macrophage polarization, supporting nerve repair.

In the peripheral nerve, exosomes contribute to axonal regeneration by supporting cellular recovery and repair (Civelek et al., 2024). Following Wallerian degeneration in PNI, EVs have been reported to increase galectin-3 expression, which facilitates myelin debris clearance, an essential step in creating a permissive environment for nerve regeneration (Wang et al., 2022b). Exosomes have also been found to upregulate the expression of cytokines and neurotrophic factors that guide axonal outgrowth: Nerve Growth Factor (NGF) and Vascular Endothelial Growth Factor A (VEGFA) were upregulated in DRGs after sciatic nerve injury, enhancing both neural and vascular regeneration (Zhao et al., 2020). Collectively, these findings highlight the ability of EVs to promote neurite outgrowth, modulate the injury microenvironment, and deliver functional biomolecules, underscoring their potential as a cell-free therapeutic modality for PNI.

## 2. Therapeutic effects of various source-derived EVs

### 2.1. Therapeutic effects of Schwann cell-derived EVs in PNI

SCs actively facilitate nerve repair by transiently degrading myelin and guiding regenerating axons to establish a pro-regenerative microenvironment (Liu et al., 2020; Saquel et al., 2022). SC-derived EVs (SC-EVs) extend these functions by delivering functionally active biomolecules that are rooted in the unique biology of SCs, which can regulate key regenerative processes promoting peripheral nerve repair. SC-EVs deliver miRNAs, such as miR-221/222 and miR-21, and proteins that regulate cytoskeletal dynamics and growth cone behavior. Delivery of these molecules have shown enhanced neurite outgrowth with directional axonal extensions and activation of survival pathways. *In vitro* studies have also shown that SC-Exosomes significantly enhance neurite outgrowth in primary SC cultures and DRG neurons, highlighting their role in axonal regeneration. The cargo within SC-Exosomes, particularly miR-221/222, was found to regulate SC migration and proliferation (Sohn et al., 2020). Additionally, SC-Exosomes can facilitate the removal of cellular debris and maintain SC homeostasis, further supporting the regenerative process after nerve damage.

A key advantage of SC-EVs is their ability to support surrounding cells. In a model using rat bone marrow mesenchymal stem cells (BMMSCs), SC-Exosomes were shown to promote the differentiation of BMMSCs into SC-like cells (SCLCs). SCLCs have a phenotype that mimics the key functional features of native SCs with increased expression of the SC markers S100 (calcium-binding proteins), Glial Fibrillary Acidic Protein (GFAP), Sex-determining Region Y-box 10 (Sox10), Nerve Growth Factor Receptor (NGFR), and Early Growth Response 2 (EGR2). These findings suggest that SC-EVs not only support axonal regeneration but also enhance tissue repair by influencing local stem cell populations (Wang et al., 2020). In another study consisting of a sciatic nerve crush injury model in Sprague Dawley rats, intraperitoneal administration of 10 μL SC-EVs from skin precursor cells (SKP-SC-EVs) at a concentration of 1 × 10^9^ particles/μL significantly improved axonal elongation across the peripheral nerve bridge after injury, with a 1.4-times greater axonal regrowth compared to the vehicle control. Furthermore, SKP-SC-EVs labeled with Protein Kinase H67 fluorescent dye (PKH67), a stain of the membrane lipid bilayer, were found to migrate directly to the injury site, activating local MSCs and further contributing to tissue regeneration (Wu et al., 2020b). These findings demonstrate that SC-EVs can enhance neuron-specific signaling which may help to promote more organized regeneration, rather than disorganized neuronal outgrowth.

Peripheral nerve regeneration depends on efficient myelin clearance and tightly regulated inflammation, promoting macrophage polarization toward a pro-regenerative phenotype. SC-EVs also help establish regenerative microenvironments that promote nerve regeneration. In an *in vivo* sciatic nerve crush injury model, SKP-SC-EVs were injected into the vitreous cavity of mice, which were shown to regulate key signaling pathways critical for neuroprotection and neuroregeneration. SKP-SC-EVs also modulated macrophage activity at the injury site, promoting myelin phagocytosis and the release of neurotrophic factors, both of which are essential for nerve healing (Cong et al., 2024). Another study showed that exosomes from lipopolysaccharide-stimulated SCs (LPS-SC-Exosomes) promoted peripheral nerve regeneration by shifting pro-inflammatory M1 macrophages to the pro-regenerative M2 phenotype. These exosomes were enriched with Ovarian Tumor Deubiquitinase with Linear Linkage Specificity (OTULIN, also known as FAM105B), a deubiquitinating enzyme which stabilizes the Erb-B2 Receptor Tyrosine Kinase 2. Local injection of 5 μL (2 μL/mL) LPS-SC-Exosomes were also shown to enhance axonal regeneration, remyelination, and functional recovery in a 3 mm rat sciatic nerve transection injury model, while OTULIN knockdown reversed these effects, highlighting the role of macrophage modulation in nerve repair (Wang et al., 2024c). This coordinated immune modulation addresses a central bottleneck in PNI: the transition from inflammation to regeneration.

In a diabetic peripheral neuropathy (DPN) model, decreased levels of miR-21 in serum exosomes correlated with impaired SC proliferation and increased apoptosis. Restoring miR-21 levels reversed these adverse effects, whereas miR-21 knockdown worsened them. Furthermore, SC-Exosomes in hyperglycemic conditions suppressed neurite outgrowth in co-cultured neurons, whereas SC-Exosomes enriched with miR-21 promoted neurite outgrowth, likely through activation of the Phosphoinositide 3-kinase/Protein Kinase B (PI3K/AKT) signaling pathway (Liu et al., 2022c). These effects, however, depend on the parent SCs’ state, as hyperglycemic conditions can also directly impair SC and exosome function. On the other hand, inflammatory support enhances their regenerative potential, highlighting that SC-Exosome activity is dynamically shaped by the parent cell microenvironment (Liu et al., 2022c).

Taken together, SC-EVs are particularly advantageous in acute injuries where timely, precise reconstruction of axonal pathways is critical. However, SC-EV therapies are limited by poor targeting specificity, rapid clearance, short circulation time, low production yield, scalability constraints, and the risk of off-target biological effects, particularly in complex diseases (Khan et al., 2025). Integrating SC-EVs with engineered components like surface functionalization for precise targeting, synthetic carriers to enhance stability and cargo loading, and biomaterial systems for controlled release may improve delivery efficiency, reproducibility, and overall therapeutic effectiveness (Khan et al., 2025).

### 2.2. Therapeutic effects of mesenchymal stem cell-derived EVs in PNI

MSCs are multipotent stromal cells derived from various sources like bone marrow, adipose tissue, muscle, and perinatal tissues. They possess unique self-renewal capacity and the ability to migrate to sites of injury, promoting repair through paracrine signaling and immunomodulation. Similarly, their extracellular vesicles (MSC-EVs) exert therapeutic effects primarily through environmental modulation rather than direct instruction of neuronal regeneration. MSC-EVs carry a rich cargo of mRNAs, miRNAs, cytokines, and growth factors which mediate intercellular communication, modulate immune responses, and support tissue regeneration (Qiu et al., 2019).

The regenerative potential of MSC-EVs is largely mediated through three interconnected mechanisms: (1) immunomodulation as a primary driver of repair, (2) indirect neurotrophic support, and (3) modulation of SC behavior. MSC-EVs reshape the injury microenvironment by suppressing T-cell proliferation and promoting regulatory T cell (Treg) expansion; polarizing macrophages to the pro-regenerative phenotype, similarly to SC-EVs; and enhancing phagocytic activity to facilitate the resolution of inflammation. MSC-Exosomes also support nerve repair through the delivery of Brain-derived Neurotrophic Factor (BDNF) and NGF both *in vitro* and *in vivo* (Cui et al., 2021; Guo et al., 2019). This positions MSC-EVs as a key regulator of the inflammatory phase, a prerequisite for successful nerve regeneration, but may not be sufficient on their own to ensure precise axonal repair, pending future controlled experiments.

MSC-EVs can influence endogenous SCs by activating signaling pathways and regulating apoptosis to enhance SC–mediated repair processes indirectly. *In vitro* studies have demonstrated that MSC-EVs significantly influence both immune cells and provide neurotrophic support: rat SC line 96 (RSC96) cells treated with 20 μg/mL MSC-EVs activated the Extracellular Signal-regulated Kinases 1 and 2 (ERK1/2) signaling pathway and showed greater phosphorylated ERK (p-ERK) expression. These changes upregulated apoptosis, eliminating damaged nerve cells and facilitating tissue homeostasis (Zhou et al., 2018). Moreover, MSC-EVs delivered neurotrophic factors that enhanced neuronal survival, axonal outgrowth, and functional repair, while also modulating SC activity through regulatory miRNAs and proteins (Chen et al., 2024). This highlights a key point that MSC-EVs can act alongside SCs to support their repair-related processes.

In a rodent 3 mm sciatic nerve crush injury model, 3 μL of human MSC-derived EVs (hMSC-EVs) loaded in a collagen gel was injected into the injured nerve 1 h after injury. This significantly reduced muscle atrophy, increased the expression of Neurofilament 200 (NF-200) and Growth Associated Protein 43 (GAP-43), and reduced apoptosis in DRGs and adjacent nerves (Demyanenko et al., 2022). These findings highlight the neuroprotective and pro-regenerative capabilities of MSC-EVs in small animal models.

**Fig. 2.**
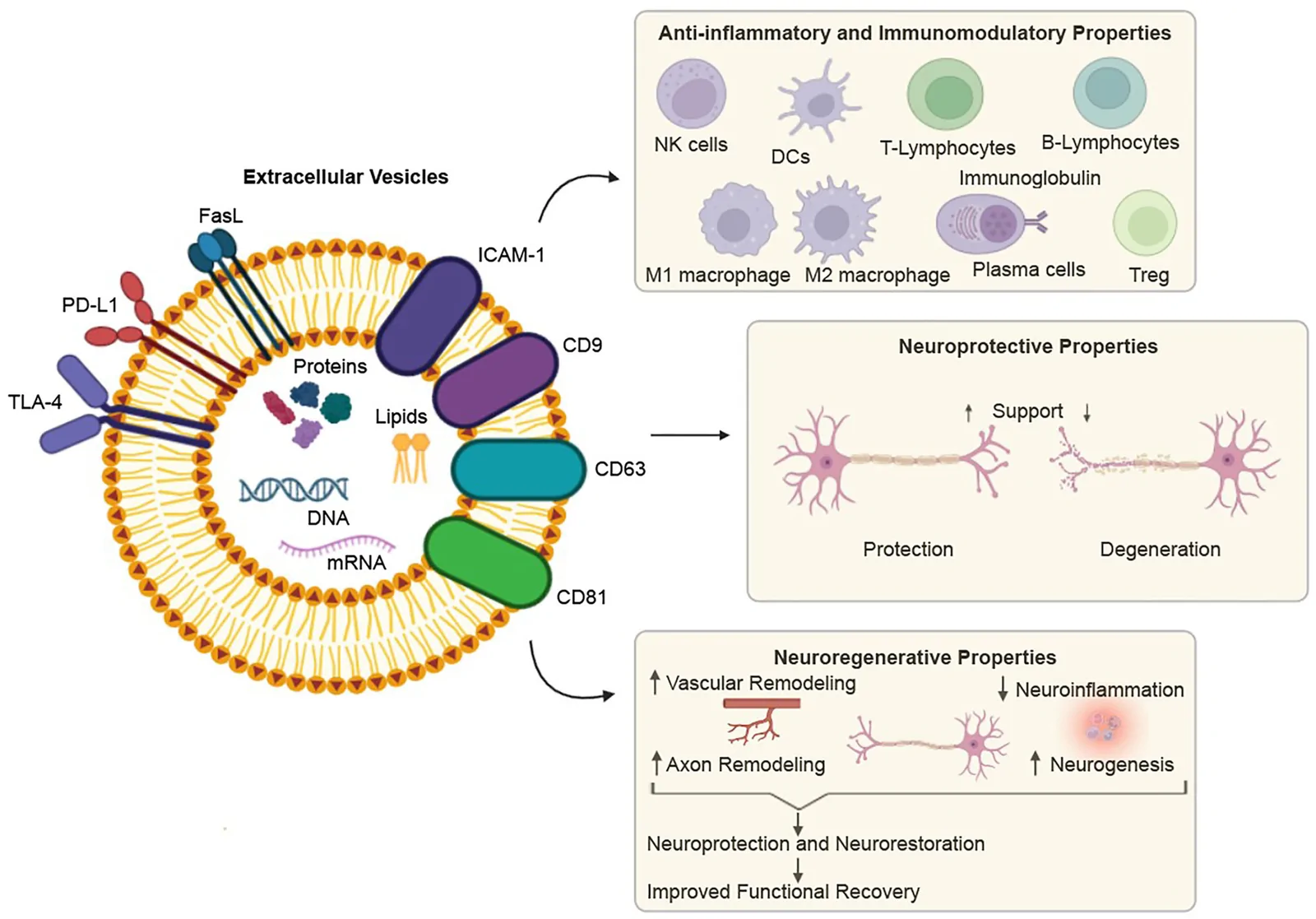
Properties of EVs as a new therapeutic strategy for PNI. EVs derived from various cell sources exert multiple therapeutic effects that support nerve regeneration after PNI. These include modulation of inflammation through the suppression of pro-inflammatory cytokines and promotion of an anti-inflammatory microenvironment; enhancement of SC proliferation, migration, and myelination; delivery of regenerative molecules such as neurotrophic factors, miRNAs, and proteins; protection of neurons from apoptosis and oxidative stress; and stimulation of axonal growth and reinnervation.

Despite their therapeutic potential, MSC-EVs face several challenges as they have a limited regenerative specificity, and their composition varies with tissue source, donor characteristics, and culture conditions. They may also improve functional recovery without fully restoring nerve structure, resulting in slower or less organized repair. As such, MSC-EVs are more effective in early-stage inflammation control and chronic injury settings, while SC-EVs are better suited for acute injuries requiring rapid, structured regeneration (Wang et al., 2024b). A detailed review of the therapeutic effects of MSC-EVs from these various sources is provided below in Fig. 2.

#### 2.2.1. Therapeutic effects of bone marrow mesenchymal stem cell-derived EVs in PNI

BMMSCs are multipotent stromal cells capable of differentiating into neural and glial-like lineages under appropriate cues. Their EVs, BMMSC-EVs, provide a promising cell-free therapeutic alternative with a uniquely reduced immunogenic risk as they lack certain hematopoietic and adhesion molecules like Grn94 (a multifunctional protein involved in neuroinflammation), CD34, and CD45, which are associated with immune activation *in vivo* (Théry et al., 2018; Tang et al., 2021). This indicates that BMMSC-EVs may possess the ability to modulate immune components of the injury microenvironment. *In vitro*, BMMSC-EVs have similarly been shown to enhance anti-inflammatory cytokine production, particularly when delivering miR-34c, significantly promoting neurovascular regeneration (Peng et al., 2023).

*In vivo* studies reinforce these mechanisms, where, in a 5 mm rat sciatic nerve crush injury model, 4 μL intramuscular (IM) injections of BMMSC-EVs significantly improved sensorimotor functional recovery over 28 days, as indicated by enhanced Sciatic Functional Index (SFI) and thermal latency scores compared to control. Histological analysis also revealed a higher density of regenerated axonal fibers and significantly larger axonal diameters in the distal sciatic nerve, confirming their neuroprotective effects (Zhao et al., 2020). In a humanized NOD scid gamma (NSG) immunodeficient mouse model of DPN, human BMMSC-Exosomes (hBMMSC-Exosomes) were co-delivered with small interfering RNAs (siRNAs) against the Fas receptor (siFas), a short hairpin RNA designed to silence the Fas cell-surface death receptor gene. 5 μL of this solution with anti-miR-375, a chemically modified oligonucleotide inhibitor of miR-375, improved cell viability under inflammatory conditions while suppressing immune activation. hBMMSC-EVs have also been found to inhibit the proliferation of peripheral blood mononuclear cells and enhance the function of Treg populations by 2.1–3.8%, highlighting their dual role in immune modulation and tissue regeneration (Wen et al., 2016). Another study on mice treated with 1 × 10^9^ BMMSC-EV particles showed improved nerve conduction velocity (NCV), decreased sensory deficits, and increased neurovascular regeneration. This was accompanied by M2 macrophage polarization and the suppression of proinflammatory cytokines, mediated at least in part by miRNAs targeting the Toll-like Receptor 4–Nuclear Factor Kappa-light-chain-enhancer of Activated B Cells (TLR4/NF-κB) signaling pathway (Fan et al., 2020). BMMSC-EVs are also rich in pre-miRNAs–such as miR-301, miR-let-7a, and miR-22–which are processed within recipient cells and function to directly increase gene expression and repair mechanisms (Kaur et al., 2018; Yi et al., 2017). These outcomes emphasized that the delivery of bioactive cargo, including growth factors, miRNAs, and pre-miRNAs, can facilitate gene regulation in recipient cells, thereby activating signaling pathways that control apoptosis, cytoskeletal dynamics, and inflammation. Critically, these outcomes suggest that BMMSC-EVs are well-characterized, scalable, and reproducible, with strong neuro-immune modulatory capacity which positions them as a promising therapy for PNI.

#### 2.2.2. Therapeutic effects of umbilical cord mesenchymal stem cell-derived EVs in PNI

Umbilical cord mesenchymal stem cells (UCMSCs), derived from Wharton’s Jelly (WJ-UCMSCs), secrete EVs rich in growth factors (BDNF, NGF), regenerative miRNAs (miR-26b, miR-133b, miR-206), and immunomodulatory molecules (Van Pham et al., 2016). UCMSC-derived EVs (UCMSC-EVs) are characterized by low immunogenicity, making them ideal candidates for allogeneic, cell-free therapies in PNI, but they also contain surface markers (CD29, CD44, CD73, CD90) and EV-specific proteins (CD9, CD63, CD81, ALG-2-interacting Protein X [ALIX]). Through these bioactive proteins, RNAs, and lipids, UCMSC-EVs have been shown to support tissue repair and neural regeneration (Monguio-Tortajada et al., 2017; Zhu et al., 2023). Their low immunogenicity, provided from their parent stem cells, and cell-free nature make them attractive candidates for allogeneic therapies in PNI.

UCMSC-EVs promote repair by modulating the injury microenvironment, enhancing SC proliferation and survival via the PI3K/AKT pathway. This helps support axonal growth, while also boosting metabolic and anabolic processes to aid differentiation and tissue regeneration. *In vitro* stimulation of WJ-UCMSCs with LPS were shown to alter the cargo composition of their derived exosomes (WJ-UCMSC-Exosomes), enhancing macrophage anti-inflammatory activity (Song et al., 2020). When 0.5 μg/μL UCMSC-Exosomes were incorporated into a 100 μg/μL concentrated hyaluronic acid methacrylate (HAMA) hydrogel system and administrated in a sciatic nerve crush injury model, macrophage infiltration was reduced, and the expression of proinflammatory cytokines such as Interleukin-1 beta (IL-1β) and Tumor Necrosis Factor-alpha (TNF-α) were decreased. Furthermore, elastic HAMA hydrogels promoted superior nerve repair compared to stiffer formulations by enabling faster exosome release, highlighting hydrogel stiffness as a key factor in optimizing exosome-based therapies for nerve regeneration (Liu et al., 2022d). These outcomes show that UCMSC-EVs, like SC-EVs, can regulate immune responses by shifting macrophages towards the M2 phenotype, thereby creating a neuroprotective, pro-regenerative milieu.

UCMSC-EVs were shown to synergize with other cell types to improve sensorimotor recovery. In a 12 mm rat sciatic nerve defect injury model, UCMSC-EVs administered as conduits with both 10 μL of 5 × 10^5^ olfactory ensheathing cells (OECs) and 10 μL EVs (400 μg/mL) per rat showed that combining OECs with EVs promoted nerve regeneration. Supporting this further, an *in vitro* experiment showed that when exosomes were incubated with OECs, there was significantly improved OEC proliferation and survival under hypoxic conditions, along with increased BDNF expression (Zhang et al., 2020). In a 5 mm rat sciatic nerve transection model, intravenous (IV) administration of 100 μg/100 μL human UCMSC-EVs (hUCMSC-EVs) promoted axonal growth, increased SC activity, improved SFI scores, and reduced muscle atrophy. Additionally, hUCMSC-EVs modulated inflammation by decreasing expression of the inflammatory cytokines IL-6 and IL-1β levels while increasing expression of the anti-inflammatory cytokine IL-10, creating a microenvironment conducive to nerve repair (Ma et al., 2019b). Similarly, IV injection of 100 μg/200 μL hUCMSC-EVs enhanced peripheral nerve regeneration after a 5 mm rat sciatic nerve bifurcation gap injury via miR-21-mediated PI3K/AKT activation (Ma et al., 2021).

The cargo of biosynthesis-related proteins in UCMSC-EVs further underscores a multilineage differentiation capacity essential for tissue repair: these proteins are known to support cellular metabolic activity and protein production, but can also enhance anabolic pathways by increasing energy generation and macromolecule synthesis (Ma et al., 2021). UCMSC-EVs share neuroprotective and immunomodulatory functions, but they also exhibit enhanced metabolic support and multilineage differentiation potential, reflecting cargo tuned to support energy-intensive regenerative processes. With low immunogenicity and straightforward isolation, UCMSC-EVs stand out as a promising, clinically translatable therapy for peripheral nerve regeneration. However, UCMSCs come from a limited biological source with high donor variability, decreasing EV yield and therapeutic consistency; therefore, EV isolation, dosing, and potency remain difficult to standardize. Additionally, although umbilical cords are ethically accessible and noninvasive to collect, proper consent, donor screening, and adherence to tissue-banking regulations are needed for safe and ethical clinical use.

#### 2.2.3. Therapeutic effects of adipose-derived mesenchymal stem cell-derived EVs in PNI

Adipose-derived mesenchymal stem cells (ADMSCs) are abundant, easily accessible, and highly stable multipotent stem cells, making them a practical source for regenerative therapies compared to other sources (Jiang et al., 2022). Subsequently, their EVs (ADMSC-EVs) inherit many of their parent cells’ neurotrophic, angiogenic, and immunomodulatory properties, providing a versatile cell-free alternative for peripheral nerve repair. A unique factor of these EVs is that, when differentiated, their EVs (ADMSC-dEVs) have refined regenerative profiles compared to their undifferentiated (ADMSC-uEVs) counterparts. *In vitro* studies using rat adrenal pheochromocytoma (PC12) cells showed that ultracentrifugation-isolated ADMSC-Exosomes significantly enhanced cell proliferation and reduced apoptosis via activation of the PI3K/AKT signaling pathway (Xie et al., 2021). In a mouse sciatic nerve crush injury model, 50 μL (1 × 10^10^ particles) of differentiated ADMSC-Exosomes (dExo)–when loaded as a pre-gel–were found to promote SC-like phenotypes and reduce oxidative stress in cultured rat SCs (Amiri et al., 2022; Liu et al., 2024). Secretome profiling of these dExo implicated beta fibroblast growth factor (β-FGF), β-NGF, Epidermal Growth Factor (EGF), EGF Receptor (EGFR), Neurotrophin-3 (NT-3), NT-4, and several other growth factors responsible for developing mature neurons (Liu et al., 2022a). Together, these factors enhance SC proliferation while also contributing to neuronal survival and axonal outgrowth.

*In vivo*, ADMSC-EVs enhance both functional and structural recovery in sciatic nerve injury models. In a 5 mm rat sciatic nerve defect model, ADMSC-Exosomes were incorporated into a biosynthetic cellulose membrane (BCM) by uniformly applying 4 μL of EV solution (1.9 × 10^8^ EV/μL) to create an ADMSC-Exosome-BCM construct. This approach promoted nerve regeneration at levels comparable to autologous nerve grafts while avoiding the donor-site complications typically associated with grafting (Cui et al., 2023). ADMSC-Exosomes enriched with miR-375 further illustrate their precision potential by downregulating Ephrin Type-A Receptor 4 (EPHA4), leading to activation of the PI3K/AKT pathway. Another study showed that, *in vitro*, miR-375 mimic-Exosomes (2 mg/100 μL) enhanced axonal outgrowth and neurotrophic factor expression in DRG neurons. Meanwhile, in rat sciatic nerve crush injury models, miR-375-enriched ADMSC-Exosomes improved functional recovery, myelinated fiber regeneration, and reduced muscle atrophy (Liu et al., 2025b).

ADMSC-EVs generally provide strong neurotrophic and axon-supportive effects, reflecting high baseline levels of neuronal growth factors and the capacity for differentiation. However, they still lack intrinsic myelin-specific factors, which may limit their ability to direct precise axonal targeting or achieve structured remyelination in acute injuries. As a broad class, MSC-EVs are ideal for chronic, complex, or inflamed injury environments, where controlling inflammation and stabilizing the microenvironment is as important as guiding individual axons. Their therapeutic potency is variable and strongly influenced by both donor characteristics and their differentiation state. As such, production standardization, cargo optimization, and dosing strategies are additional key challenges that must be overcome before their human translation for PNI.

### 2.3. Therapeutic potential of EVs derived from other stem cells in PNI

EVs originating from dental pulp stem cells (DPSC-EVs) and human induced pluripotent stem cells (hiPSC-EVs) demonstrate promising neuroregenerative effects in PNI, but their mechanisms and specificity differ from the previously discussed SC-EVs and MSC-EVs. DPSC-Exosomes carry miR-122–5p, which suppresses P53 expression in SCs and prevents excessive autophagy after nerve injury, maintaining a balanced regenerative environment. Inhibition of DPSC-Exosome release and overexpression of P53 reverses these effects, underscoring the precision of DPSC-Exosomes in targeting specific molecular pathways to enhance myelin repair (Chai et al., 2024). DPSC-EVs also attenuate pro-inflammatory macrophage polarization through the Mitogen-Activated Protein Kinase (MAPK)–NFκB-P65 pathway, highlighting a dual neuroprotective and anti-inflammatory function that indirectly facilitates axonal regeneration. In an *in vitro* injury model, 100 ng/mL of LPS was used to injure RAW264.7 cells and induce reactive oxygen species (ROS) production. When treated with 10 μg/mL of DPSC-Exosomes, however, MAPK-NFκB-P65 signaling was upregulated and M1 macrophage polarization was subsequently inhibited (Liu et al., 2022b). These mechanistic insights suggest that DPSC-EVs act as focused modulators of SC behavior and immune responses, offering targeted intervention for PNI.

hiPSC-EVs provide broad neurotrophic and regenerative support by delivering a diverse cargo of neurogenic miRNAs and proteins (Upadhya et al., 2020). A rat sciatic nerve crush injury model showed that local treatment with 10 μL (1.1 × 10^11^ particles/mL) of hiPSC-Exosomes enhanced axonal regeneration and functional recovery, as evidenced by improved gait and strength scores, with RNA sequencing revealing activation of the PI3K-AKT and focal adhesion signaling pathways (Aldali et al., 2025). These studies collectively highlight iPSC-EVs’ ability to create an extensive pro-regenerative environment, rather than executing highly targeted structural guidance, suggesting they are most effective as broad neuroprotective agents.

### 2.4. Therapeutic potential of EVs derived from non-stem cell sources in PNI

Beyond stem cell sources, EVs can be derived from non-stem cells–such as fibroblasts and vascular endothelial cells–which also exhibit strong regenerative potential in PNI. Fibroblast-derived exosomes (f-Exosomes) have been shown to promote neurite outgrowth across various neuronal types, including cortical neurons, DRG neurons, and retinal ganglion cells, even in unfavorable inhibitory environments (Tassew et al., 2017). Preconditioning fibroblasts with the naturally derived polymer chitosan oligosaccharides (COS-f) has been shown to enhance their secretion of exosomes enriched with Transcription Factor AP-2 Gamma; this family of transcription factors regulates development and differentiation by suppressing miR-132–5p in DRG neurons. This suppression upregulates Calcium/Calmodulin-Dependent Protein Kinase 1 (CAMKK1), a serine/threonine kinase that promotes neurite outgrowth and axon regeneration both *in vitro* and *in vivo*. In a 10 mm rat sciatic nerve defect model, COS-f-Exosomes (8.1 × 10^8^ particles/rat) and a miR-132–5p antagonist (5 nmol/rat) were shown to upregulate CAMKK1 signaling, promoting axonal regeneration and functional recovery (Zhao et al., 2023).

Vascular endothelial cell-derived exosomes (VEC-Exosomes) have also demonstrated neuroprotective effects by reducing apoptosis and promoting the proliferation of neural progenitor cells (NPCs) following an *in vitro* ischemic injury model. By enhancing NPC proliferation, these exosomes directly regenerate lost or damaged neural tissue (Zhou et al., 2020). In a separate study, EVs isolated from neurons co-cultured with ADMSCs *in vitro* were found to carry a unique cargo profile: Synaptosomal-associated Protein, 25 kDa, miR-132, and miR-9. Together, these regulatory molecules enhanced neuronal development and synaptic function in mice (Roballo et al., 2019). EVs derived from neural crest cells (NCC-EVs) were found to also carry neurotrophic factors, like NGF, at a concentration of 1 × 10^9^ particles/mL in Balb/C female mice; this promoted axonal regeneration, SC activation, and remyelination *in vitro*. NCC-EVs also suppressed oxidative stress–induced neuronal apoptosis and the production of pro-inflammatory cytokines (Yeo et al., 2025). Altogether, the regenerative effects of these alternative stem cell EVs (DPSC-EVs, hiPSC-EVs) and non-stem cell EVs (f-EVs, VEC-EVs, NCC-EVs) are driven by specific molecular signaling mechanisms that facilitate diverse regenerative functions, including the promotion of neurite outgrowth, axonal regeneration, myelin repair, and immunomodulation. A summary of EV sources, types, and their regenerative properties is provided in Table 2.

### 2.5. Summary comparison of EV sources

EVs from different stem cell sources exhibit distinct functional profiles that position them within complementary mechanistic niches for PNI therapy. SC-EVs provide axonal guidance and promote remyelination effectively orchestrating nerve regenerative processes (Jang et al., 2017). In contrast, BMMSC-EVs, ADMSC-EVs, and UCMSC-EVs exert broad trophic and immunomodulatory effects to create a favorable microenvironment that suppresses inflammation (Cong et al., 2024; Cui et al., 2024; Feng et al., 2019). f-EVs and NCC-EVs deliver targeted pro-regenerative signals (Yeo et al., 2025; Zhao et al., 2023), while DPSC-EVs modulate SC autophagy and local immune responses to promote myelin repair (Liu et al., 2022b). hiPSC-EVs also deliver strong neuroprotective and regenerative signals across multiple pathways, supporting non-specific regeneration and functional recovery in complex injury environments (Lu et al., 2025).

## 3. Pharmacological properties of EVs

### 3.1. Anti-inflammatory and immunomodulatory properties of EVs

While neuroinflammation is necessary for the body’s early defense after PNI, it can also delay recovery if prolonged. EV-based therapies have shown promise in managing this response by targeting key neuroinflammation regulators, such as NF-κB, to reduce pro-inflammatory cytokine expression and foster a more regenerative microenvironment (Gu et al., 2024). Exosomes derived from MSCs–including those from ADMSCs and BMMSCs–are naturally enriched with a variety of functional proteins like tetraspanins and integrins (Gholami Farashah et al., 2022; Wei et al., 2024). Furthermore, MSC-Exosomes carry proteins essential for membrane fusion, such as the Ras superfamily of small GTPases, annexins, and heat shock proteins (HSP70 and HSP90). Similar to other exosomes, these proteins are source-dependent: for example, ADMSC-Exosomes contain significantly higher levels of enzymatically active neprilysin compared to BMMSCs, permitting greater proteolytic clearance of bioactive peptides (Katsuda et al., 2013).

Beyond their protein makeup, the clinical value of MSC-EVs lies in their ability to rebalance immune signaling. They can dampen the inflammatory storm by downregulating pro-inflammatory cytokines such as IL-1β, IL-6, TNF-α, and IL-12p40, while promoting the secretion of anti-inflammatory cytokines like IL-10 and Transforming Growth Factor beta 1 (TGF-β1) (Bazzoni et al., 2020; Wang et al., 2025c). This influence extends to the microenvironment as well, where MSC-EVs direct a variety of soluble mediators like Interferon-gamma (IFN-γ), Prostaglandin E2 (PGE2), and Heme Oxygenase-1 (HO-1). These coalesce with stimulation of the Signal Transducer and Activator of Transcription 3 (STAT3) pathway, which suppresses NF-κB signaling, underscoring the therapeutic potential of MSC-EVs in treating neuroinflammatory conditions and enhancing nerve repair (Rao et al., 2024).

While MSC-EVs can provide broad-spectrum immunomodulation, SC-EVs appear to offer a more targeted effect that directly benefits neuronal regeneration. Following PNI, SCs naturally recruit immune cells, such as macrophages, to the injury site and their EVs shift the receptor profile of regenerating cells from TNF Receptor (TNFR) 1, which triggers apoptosis, to TNFR2, which promotes pro-survival signaling pathways, to enhance the nerve’s regenerative potential (Beldi et al., 2020). Such findings highlight how EVs are not interchangeable but instead occupy distinct immunomodulatory niches that work synergistically in PNI repair.

### 3.2. Neuroprotective properties of EVs

The neuroprotective capacity of EVs stems from a combination of anti-apoptotic, pro-angiogenic, anti-oxidative, and stress-response regulatory mechanisms, many of which are mediated by miRNA cargo (Fig. 2). A critical precursor for nerve repair is the timely restoration of blood supply. Studies have shown that miR-125a, carried by ADMSC-Exosomes, can inhibit the expression of Delta-like ligand 4, thereby promoting the formation of endothelial cells and capillaries (Liang et al., 2016). These exosomes can also inhibit apoptosis by upregulating the anti-apoptotic marker B-cell Lymphoma 2 (Bcl-2) and downregulating the pro-apoptotic marker Bcl-2-associated X Protein (Bax) in SCs. This neuroprotective effect is mediated via endocytosis, where ADMSC-Exosomes are internalized by SCs and trigger these gene expression changes without directly binding or fusing with the cell membrane (Liang et al., 2016; Wang et al., 2016). Another ADMSC-EV miRNA cargo, miR-25–3p, was shown to reduce autophagy in neurons, a process that can otherwise exacerbate neuronal injury if left unchecked (Kuang et al., 2020). In a separate study, endothelial progenitor cell-derived exosomes can carry miR-137, which provided protection against oxyhemoglobin-induced toxicity in a human neuroblastoma cell line (SH-SY5Y) through the Cyclooxygenase-2 (COX-2)/PGE2 pathway (Li et al., 2020b). Furthermore, exosomes carrying miR-92b-3p can help neurons navigate oxygen-glucose deprivation (OGD) conditions by regulating cellular stress responses (Roballo et al., 2019; Xu et al., 2019a). This is of particular importance for neural damage in ischemic conditions, where exosomes from various stem cells, such as ADMSCs and M2 microglia, can reduce ROS formation. Exosomes from the latter, in particular through the secretion of miR-124, inhibits neuronal apoptosis induced by OGD, thereby providing a neuroprotective effect by counteracting oxidative stress and enhancing cell survival (Xie et al., 2023a).

BMMSC-EVs promote neuronal survival and regeneration through diverse mechanisms, making them an attractive candidate for clinical translation. Exosomes derived from hypoxia-preconditioned BMMSCs protect neural cells against OGD-induced injury by suppressing Pyrin Domain-containing 3 (NLRP3) inflammasome-mediated pyroptosis, a highly inflammatory form of cell death. Furthermore, BMMSC-Exosomes can also modulate microglial polarization, promoting the anti-inflammatory M2 phenotype which reduces neuroinflammation and increases tissue repair following ischemic injury (Liu et al., 2021). Beyond miRNAs, exosomes are a sophisticated delivery vehicle for a myriad of neurotrophic factors that are pivotal for the survival and growth of neurons. These include, but are not limited to, FGF, NGF, Ciliary Neurotrophic Factor, BDNF, and Glial Cell Line-derived Neurotrophic Factor (GDNF) (Bucan et al., 2019; Li et al., 2020a; Nemati Mahand et al., 2024). A critical but often overlooked aspect of exosome-mediated neuronal protection is their ability to maintain metabolic homeostasis and mitochondrial health. Following *in vitro* ischemic injury, neurons and astrocytes are susceptible to calcium overload, a significant cause of mitochondrial ROS production and cell death. By buffering intracellular Ca^2+^ levels, exosomes can preserve mitochondrial function and directly counteract such oxidative stressors (Yin et al., 2025). Some of these key neuroprotective mechanisms coalesce at the PI3K/AKT signaling pathway, which promotes cell survival while inhibiting apoptosis (Liu et al., 2025a; Wang et al., 2025b). This suggest that BMMSCs particularly excel at stimulating this protective pathway while conferring multifaceted trophic support and suppressing inflammatory signals.

Through their role as targeted gene-regulatory delivery systems, EVs coordinate neuroprotection at multiple levels, including the suppression of pyroptosis, modulation of microglial polarization toward the reparative M2 phenotype, and delivery of neurotrophic factors that activate pro-survival pathways. Together, these mechanisms underscore a division of labor in which MSC-derived EVs provide broad cytoprotective effects.

### 3.3. Neuro-regenerative properties of EVs

Beyond protection, EVs actively drive axonal and neural regeneration after PNI. SCs play a central role in this process by not only supporting growth cone modulation but also guiding the migration of regenerating axons. Their EVs can inhibit RhoA GTPases, key mediators of growth cone collapse and axon retraction, which allows regenerating axons to extend filopodia and lamellipodia to explore the extracellular environment and promote axonal extension (Tan et al., 2018).

As with neuroprotection, miRNAs also have profound neuro-regenerative capabilities. One such miRNA is miRNA-21, which has been shown to target Sprouty2, a negative regulator of the Ras/ERK signaling pathway, and promote axonal regrowth in DRG neurons following PNI (Strickland et al., 2011). These exosomes, when internalized by neurons, can then downregulate Phosphatase and Tensin Homolog (PTEN) and activate the PI3K/AKT signaling pathway (López-Leal et al., 2020). Another study further supports this mechanism, highlighting that miRNA-21-enriched EVs enhance sensory neuron growth and survival by negatively regulating PTEN (Saquel et al., 2022). Furthermore, the critical role of miRNA-mediated gene regulation in peripheral nerve repair is highlighted by studies showing that deletion of Dicer, an essential miRNA-processing enzyme, in injured neurons significantly impairs axonal growth and functional recovery (Viader et al., 2011). These findings represent miRNA-21-enriched EVs as a central and transferable regenerative axis. In a tangential signaling pathway, BMMSC-derived EVs can also facilitate axonal regeneration by mobilizing autocrine Wnt10b, a glycoprotein involved in the Wnt/β-catenin pathway. Prior studies found that after f-Exosome treatment, Wnt10b is recruited to lipid rafts, activating mechanistic/mammalian Target of Rapamycin (mTOR) via Glycogen Synthase Kinase 3 beta and Tuberous Sclerosis Complex 2 (TSC2), enhancing the intrinsic regenerative capacity of neurons (Tassew et al., 2017). These f-Exosomes can also carry miR-126 and miR-21–5p to exert similar effects via PI3K/AKT and MAPK/ERK signaling.

Apoptosis of SCs following nerve injury significantly impedes the regenerative process. Accordingly, ADMSC-Exosomes can limit ROS formation by releasing Nicotinamide Adenine Dinucleotide Phosphate Oxidase 2 (NOX2) complexes into injured axons. This process is mediated through the NOX2-PI3K-p-AKT signaling pathway, and permits a favorable environment for SCs to proliferate and support myelin repair (Liu et al., 2020). Since such regrowth requires vast stores of neurotrophic factors, MSCs–such as ADMSCs–can derive exosomes that contain relatively high concentrations of BDNF, FGF, GDNF, and NGF to enhance neuro-regeneration (Bucan et al., 2019; Xie et al., 2021). *In vivo*, BMMSC-Exosomes preserve the structural integrity of nerve bundles; these regenerative effects are attributed to the miRNA-mediated regulation of genes such as Peripheral Myelin Protein 22, VEGFA, NGFR, and S100 Calcium-binding Protein B (Zhao et al., 2020). As such, EV-mediated neuroregeneration is not a uniform process, but reflects clear source-dependent mechanistic specialization with distinct functional trade-offs.

Taken together, SC-EVs drive axonal guidance through RhoA inhibition and growth cone stabilization, while their miR-21 cargo activates key regenerative pathways such as PI3K/AKT and ERK. In parallel, BMMSC-EVs enhance intrinsic neuronal growth via Wnt10b–mTOR signaling, and ADMSC-EVs optimize the regenerative microenvironment by reducing oxidative stress and supporting SC survival. These observations support a model in which effective nerve repair likely requires context-specific EV strategies tailored to injury type, stage, and micro-environmental constraints.

## 4. Impact of EVs on neuronal pathways

### 4.1. TSG-6/NF-κB/NLRP3 signaling pathway

**Fig. 3.**
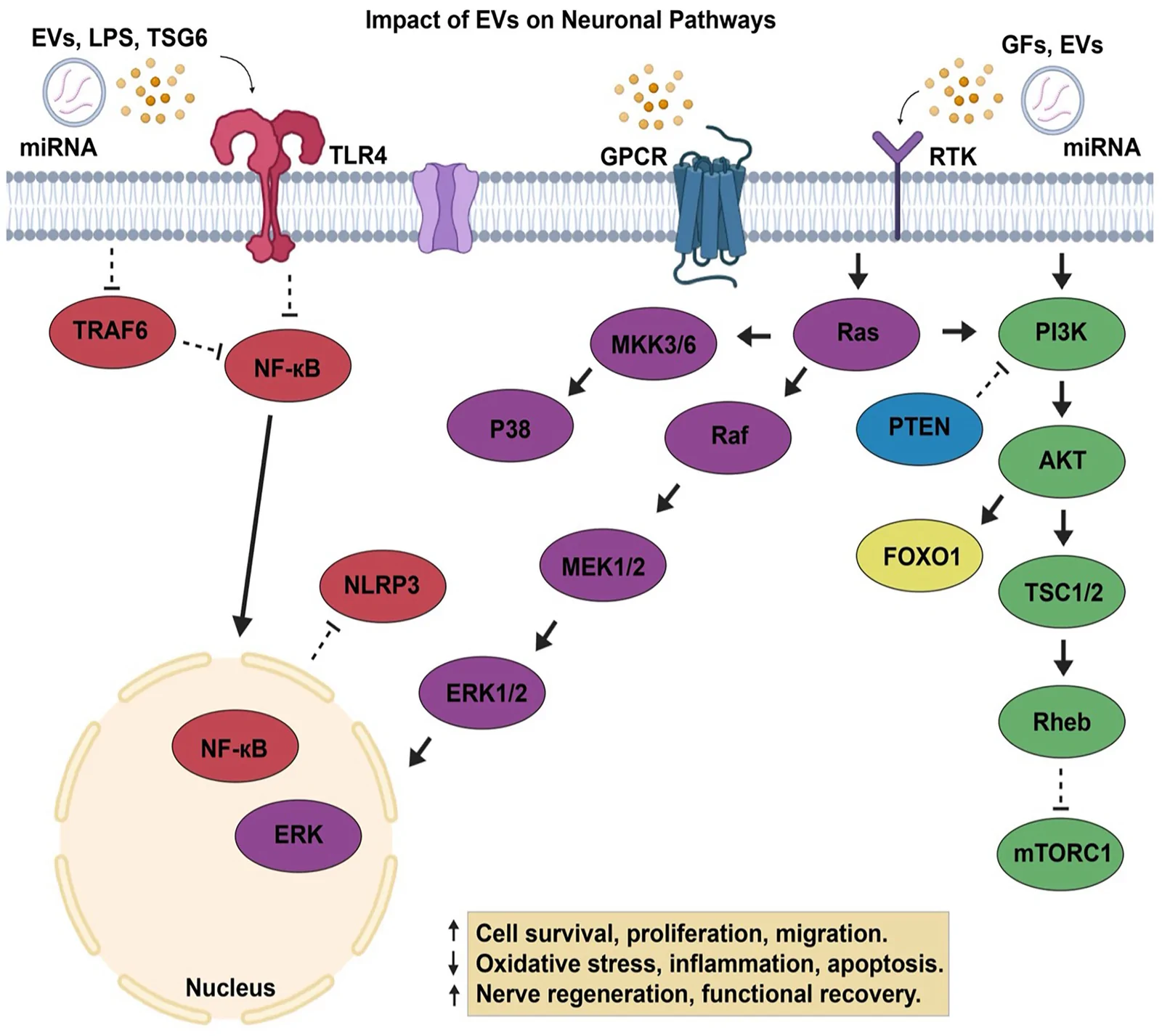
EVs impact various nerve regulatory pathways involved in PNI and repair. These include pathways regulating inflammation (TLR4, NF-κB, NLRP3/NLRP6), SC activation and myelination (MEK/ERK/MAPK, PI3K/AKT/PTEN), axonal growth and cytoskeletal dynamics (mTOR), and apoptosis and oxidative stress responses. By delivering bioactive cargo such as miRNAs, proteins, and lipids, EVs fine-tune these pathways to promote neuronal survival, enhance SC phenotype transitions, stimulate axonal elongation, and facilitate functional recovery.

The TNF-stimulated Gene 6 (TSG-6)/NF-κB/NLRP3 axis controls the inflammatory microenvironment after PNI. Excessive activation of NF-κB triggers M1 macrophage polarization and NLRP3 inflammasome formation (Fig. 3), leading to high levels of IL-1β, IL-6, and TNF-α. This pro-inflammatory state has multiple detrimental effects on nerve repair. Elevated cytokine levels induce neuronal apoptosis by activating caspase pathways, thereby reducing the number of surviving neurons and limiting the pool of axons available for regeneration. At the same time, chronic inflammation impairs SC function, disrupting their proliferation and myelination capacity, slowing axonal regrowth. Furthermore, persistent inflammatory signaling promotes tissue scarring and oxidative stress, leading to fibrosis and accumulation of ROS, which together create physical and chemical barriers that can hinder axon extension and the overall regenerative process. When preconditioned with LPS (LPS-pre-Exosomes) and enriched in miR-222–3p, these exosomes can mediate immunomodulatory functions through the NF-κB/NLRP3 signaling axis, promoting M2 macrophage polarization (Zhang et al., 2023). By modulating these pathways, LPS-pre-Exosomes mitigate the inflammatory response and support a more favorable environment for nerve repair and regeneration. Similarly, SC-Exosomes promote axon regeneration and remyelination by directly targeting neurons and myelin-forming cells. SC-Exosomes also regulate macrophage-mediated inflammatory infiltration following PNI by downregulating miR-146a-5p, which inhibits the TNF Receptor-associated Factor 6 (TRAF6)/NF-κB pathway after injury (Li et al., 2022a; Sun et al., 2023). These factors improve motor recovery, preserve neurons, and enhance axon–SC interactions, enabling effective muscle reinnervation. In rat PNI models, this leads to better gait coordination, higher SFI scores, increased compound muscle action potential (CMAP) amplitudes, and greater muscle weight. This indicates that MSC EVs preconditioned macrophages toward the global inflammatory shift by suppressing NF-κB/NLRP3 signaling.

### 4.2. MAPK/ERK signaling pathway

Growth factor–mediated activation of receptor tyrosine kinases triggers the MAPK/ERK (MEK) pathway (Fig. 3), which enhances axonal transport, protein synthesis, and the assembly of microtubules and actin filaments to mediate neuro-regeneration (Hausott and Klimaschewski, 2019). While the activation of MAPK is crucial for the development and persistence of PNI-induced neuropathic pain (Fig. 3), both ERK and p38, key members of the MAPK family, contribute to various physiological and pathological processes, including inflammation, cellular proliferation, differentiation, and apoptosis (Huang et al., 2022).

Electroconductive exosomes (ECH-Exosomes) are BMMSC-Exosomes combined with materials that enable electrical conductivity and have been shown to enhance myelinated axonal regeneration via the MEK pathway both *in vitro* and *in vivo*. This effect ameliorates muscle denervation atrophy and promotes functional restoration, as evidenced by increased CMAP amplitudes and SFI scores (Yang et al., 2023). Although ERK is not the primary mediator of neuronal survival during development, its activation is essential for the postnatal survival of nociceptive sensory neurons, particularly in response to stress or toxicity (Hausott and Klimaschewski, 2019; Huang et al., 2022). Additionally, activation of the ERK pathway via BMMSC-Exosomes has been shown to promote nerve regeneration following facial nerve injury, and inhibition of the ERK pathway has been found to impair facial nerve regeneration, highlighting its critical role in the repair process (Sun et al., 2017). This mechanistic link explains how molecular ERK activation translates to observable recovery of motor function *in vivo*, indicating that BMMSC-EVs are a potential activator of MEK signaling to drive robust regeneration and remyelination.

### 4.3. PI3K/AKT/mTOR signaling pathway

The PI3K/AKT/mTOR axis is a major regulator of neuronal survival, axon elongation, SC support, and angiogenesis (Iranpanah et al., 2023). As previously mentioned, EVs derived from stem cells and endothelial cells have been shown to activate this pathway, primarily through growth factors and miRNA cargo. Central to this effect is mTOR Complex 1, a complex regulated by PI3K/AKT activity via inhibition of the TSC1/TSC2 or activation of the Ras Homolog Enriched in Brain complexes, respectively (Fig. 3). Activation of PI3K/AKT/mTOR by EVs can also reduce oxidative stress, apoptosis (Bax/Bcl-2, caspase-3), and inflammatory signaling (NF-κB) (Fig. 3) (Fakhri et al., 2021; Wu et al., 2020b).

EVs derived from SKP-SCs increase phosphorylation of AKT, mTOR, and p70 Ribosomal S6 Kinase in motor neurons, and pre-treatment with resveratrol further enhances autophagy and apoptosis regulation. Although this interaction may depend on additional modulatory factors, it translates into improved motor function via the PI3K/AKT/mTOR axis (Wu et al., 2020b). MSC-Exosomes similarly promote neuroprotection via PI3K/AKT activation (Xie et al., 2021). Additionally, endothelial cell-derived exosomes (EC-Exosomes) upregulate pro-survival genes to increase PI3K/AKT phosphorylation and downregulate PTEN (Fig. 3), thereby enhancing axon regeneration and SC repair phenotypes (Huang et al., 2023); these genes include Growth Differentiation Factor 15, Cellular Communication Network Factor 1, Member of Activator Protein-1, Thioredoxin-interacting Protein, and Krüppel-like Factor 10, which were all upregulated following treatment with EC-Exosomes (Huang et al., 2023). This suggests that EC-Exosomes are strongly involved in the phenotypic transformation and activation of the PI3K/AKT survival signaling pathway (Fig. 3), ultimately playing a key role in the regeneration and re-establishment of neuronal function. By boosting SC repair-related phenotypes, EV-based therapies can contribute directly to nerve regeneration (Wang et al., 2020, 2024a).

Low-intensity pulsed ultrasound (LIPUS)-stimulated SC-Exosomes and ADMSC-Exosomes enhance PI3K/AKT activity to promote SC differentiation, as well as proliferation and anti-apoptotic responses (Ye et al., 2023). Mechanistically, SC-Exosomes activate the PI3K/AKT/-Forkhead box O (FOXO) axis to regulate transcriptional programs involved in stress resistance and repair (Wang et al., 2020; Ye et al., 2023). In parallel, ADMSC-Exosomes potentiate the differentiation of ADMSCs into SC–like phenotypes, demonstrating a 2.1-fold upregulation of PIK3CD and increased levels of phosphorylated AKT. Beyond differentiation, ADMSC-Exosomes enhance other key regenerative processes: in rat PC12 cells, purified ADMSC-Exosomes significantly increased the proliferative and migratory capacity of these cells while reducing apoptotic rates. These effects were abolished by the PI3K/AKT inhibitor LY294002, confirming pathway dependence (Xie et al., 2021). These molecular and cellular effects support neuronal survival and SC-mediated repair, both of which are critical for axonal regeneration and remyelination.

EC-Exosomes activate the PI3K/AKT/PTEN signaling pathway to drive the repair phenotypes of SCs. *In vivo* studies showed that EC-Exosomes have a strong neuronal affinity, persisting in nerve tissue for extended periods. EC-Exosomes can also modulate functional recovery in rats with sciatic nerve crush injury, as demonstrated by improvements in SFI, CMAP, and gastrocnemius muscle weight ratio; immunofluorescent staining and transmission electron microscopy further confirmed this beneficial effects of EC-Exosomes on nerve repair (Huang et al., 2023). By connecting intracellular signaling to structural and functional outcomes, these studies illustrate that EV-induced PI3K/AKT/mTOR activation can mechanistically drive behavioral and electrophysiological improvements following PNI (Fig. 3). Overall, EC-EVs can act as potential activators of the PI3K/AKT/mTOR pathway, driving sustained AKT signaling, PTEN suppression, and pro-survival gene expression that supports nerve regeneration.

### 4.4. Other signaling pathways

Beyond the major signaling axes, different source derived EVs have been engaged in a diverse network of signaling cascades to orchestrate neuroprotection, immunomodulation, and regenerative processes. ADMSC-Exosomes (50 ng/mL) were reported to suppress LPS-induced cytotoxicity in neural injury models by downregulating inflammatory markers (TNF-α, IL-1β, IL-6, COX-2, inducible Nitric Oxide Synthase [iNOS]) and other signaling molecules (p-P38, p-P65, p-ERK, and p-JNK) to block NF-κB and MAPK pathways in SH-SY5Y and BV-2 cells (Feng et al., 2019). This coordinated suppression of inflammatory signaling reduces neuronal damage and preserves the cellular environment required for proper regeneration. MSC-EVs further exert immunomodulatory effects through their miRNA cargo. For example, miR-21 and miR-181c suppress NF-κB and TLR4 signaling, while miR-21–5p promotes M2 macrophage polarization; additional miRNAs (miR-326, miR-182, miR-17–5p, miR-140–5p, miR-9, miR-let7) further contribute to the downregulation of pro-inflammatory cytokines (Das et al., 2014). Beyond inflammation, these exosomes can regulate oxidative stress pathways (Nuclear Factor Erythroid 2–related Factor 2 [Nrf2]/HO-1, Superoxide Dismutase [SOD], glutathione), as well as apoptosis (Bax, Bcl-2, caspases) and matrix remodeling pathways. They are also able to engage in greater interconnected signaling networks such as MAPK, CREB/BDNF, GSK-3β, and JAK/STAT (Cui et al., 2021, 2024).

In summary, EVs reduce inflammation and oxidative stress while enhancing SC survival, proliferation, and neurovascular support to facilitate axonal elongation, remyelination, and synaptic reestablishment. Altogether, MSC-Exosomes provide broad immunomodulatory and cytoprotective effects, ADMSC-Exosomes emphasize anti-apoptotic and proliferative support, and SC-Exosomes specialize in neuron–axon communication. Collectively, this indicates that MSC-EVs modulate multiple signaling pathways through miRNA cargo, while ADMSC-EVs can also promote anti-inflammatory and anti-apoptotic effects via MAPK/NF-κB suppression (Fig. 3). The convergence of multiple signaling pathways underscores both the versatility and the source-dependent roles of EVs, suggesting that their successful clinical translation will likely require leveraging these multiple regenerative pathways. These studies highlight a clear source-dependent specialization of EV function, where different EV types dominate specific signaling axes (Cui et al., 2024; Feng et al., 2019; López-Leal et al., 2020), however direct comparative mechanistic studies evaluating how EVs from different sources activate distinct signaling pathways remain limited and should be prioritized in future research.

## 5. EVs as natural nanocarriers

Nanotechnology has emerged as a promising therapeutic strategy for various biomedical applications and offers a suite of multifunctional platforms including micelles, liposomes, mesoporous silica nanoparticles, microneedles, polymersomes, and emerging 2-dimensional inorganic nanosheets that can be engineered for enhancing drug delivery. However, each carries significant trade-offs: for example, liposomes are able to engage the immune system and remain active in circulation for extended periods, but are limited by poor degradation and potential toxicity (Amiri et al., 2022). In contrast, exosomes forgo this trade-off, instead combining the benefits of synthetic nanoparticles, such as nanoscale size, passive targeting, enhanced permeability, and retention effect, with unique biological advantages like low immunogenicity, intrinsic targeting capacity, and non-cytotoxicity. These properties alone make EVs a worthwhile alternative for exploring various clinical applications (Amiri et al., 2022).

As a biological delivery system, EVs are enriched with native membrane proteins and carrier ligands, including tetraspanins and integrins, which can be used to facilitate targeted drug delivery by binding to receptors with high specificity. The presence of a wide array of integral and peripheral membrane proteins on EVs further enhances their potential in therapeutic applications compared to other drug delivery systems. These include neuroregeneration and neuroprotection, with particularly high penetration through blood-nerve barriers. Cytophilic and homing selectivity properties, along with minimal cytotoxicity, make EVs or EV mimetics ideal candidates for drug delivery systems. As such, drug-loaded EVs represent a rapidly growing area in the medical and pharmaceutical fields. For example, ADMSC-Exosomes loaded with NT-3, a prominent neurotrophic factor often deficient in early-stage PNI, restored regenerative support in a rat sciatic nerve 10 mm defect model. When embedded in an alginate hydrogel conduit, these exosomes significantly improved gastrocnemius muscle function and motor recovery, demonstrating the translational potential of exosome-based bioengineered therapies (Yang et al., 2021). In mouse DPN models, miR-146a-loaded BMMSC-Exosomes administered IV weekly for 8 consecutive weeks at 1 × 10^9^ particles/animal, were found to decrease the threshold for thermal and mechanical pain stimuli, while increasing NCV (Fan et al., 2020). In another study, SKP-SC-EVs transfected with miR-21–5p significantly enhanced neurite growth in high glucose conditions compared to EVs derived from glucose-deprived SCs through regulation of the p-AKT signaling pathway (Cong et al., 2021). Moreover, BMMSC-Exosomes with polypyrrole nanoparticles (PpyNPs)-loaded liposomes were injected IM into DPN rats, which improved CMAP and NCV, highlighting the potential of exosomes as multifunctional nanocarriers for neural repair (Singh et al., 2021a). A summary of EVs as nanocarriers loaded with mRNA and proteins is provided in Table 3.

A variety of methods have been developed for loading therapeutic agents into EVs, which are broadly categorized into physical, chemical, and biological approaches. Physical loading methods apply external forces to transiently disrupt the exosome membrane, facilitating cargo incorporation (Wang et al., 2022a). Techniques such as sonication use high-frequency sound waves to permeabilize membranes, enabling the encapsulation of both hydrophilic and hydrophobic molecules though potentially at the cost of vesicle integrity (Du et al., 2021). Similarly, freeze-thaw cycles, while straightforward and economical, can lead to vesicle aggregation and reduced bioactivity (Görgens et al., 2022). Another method, extrusion, forces exosomes and their cargo through nanoscale pores and ensures uniform vesicle size, but this method may also compromise membrane stability (Sun et al., 2021). Meanwhile, electroporation applies an electric field, forming temporary pores in EV membranes to allow entry of large or charged molecules, though it risks EV aggregation and degradation of sensitive cargo (Rong et al., 2023).

Chemical loading methods involve the use of membrane-modifying agents or physicochemical gradients. For instance, saponin-assisted permeabilization exploits saponin’s interaction with cholesterol to create transient pores, providing high loading efficiency, albeit with the need to remove residual surfactants to prevent toxicity (Haney et al., 2015). Another strategy, pH gradient loading, enhances the encapsulation of nucleic acids without significantly altering vesicle size or charge, though stability concerns remain (Jeyaram et al., 2020). Lastly, biological approaches harness endogenous EV loading mechanisms. Transfection of donor cells allows therapeutic molecules to be naturally packaged into EVs during biogenesis. While this offers specificity, it also presents the risk of altering EV composition (Yu et al., 2022). Alternatively, simple incubation with cargo enables passive diffusion into vesicles under optimized conditions; this method preserves exosome structure but may yield lower loading efficiencies, particularly for hydrophilic compounds (Wang et al., 2022a). Collectively, the choice of loading method depends on the physicochemical properties of the cargo, the desired therapeutic outcome, and the required preservation of exosomes integrity and functionality.

Among these, physical and chemical methods are more commonly used due to their simplicity and high encapsulation efficiency; meanwhile, biological methods–though specific–tend to be slower and less efficient. Successful drug loading depends on optimizing physical membrane-cargo interactions, loading parameters (e.g., force, time, temperature), and concentration gradients based on cell type. Therefore, the selection of an appropriate drug loading method is essential to ensure effective target molecule conjugation, EV integrity, and therapeutic outcomes.

## 6. Bioengineered EVs

Bioengineered EVs offer unprecedented versatility by allowing customization of both EVs and their cargo molecules. Engineering strategies for EVs include modifying the production and function of parent cells using various biochemical factors, such as pro-inflammatory mediators, growth factors, and nanoparticle formation. These approaches involve modifying the parent cells or directly altering the EVs themselves to optimize their regenerative functions. While engineered EVs play critical roles in enhancing regenerative repair, reducing inflammation, and maintaining physiological homeostasis, the natural biofunction and yield of engineered EVs are inherently limited due to inconsistencies between batches and off-target effects. This highlights the need to engineer both source cells and EVs themselves to maximize their therapeutic potential in the PNS (Jiang et al., 2025). Several engineering approaches have been employed to improve the properties of EVs (Jiang et al., 2025; Richter et al., 2021): (1) Genetic modification, which includes transfection of a plasmid carrying a specific protein of interest into the host cells, followed by transduction, (2) Biochemical conjugation, which involves chemical coupling and bonding of functional ligands, hydrophobic inclusion sites, small molecules peptides, or aptamers on EV surfaces to improve recognition and binding, (3) Nanomaterial incorporation, which integrates magnetic metals or organic polymetric nanosheets either within the EV lumen or on its surface, and (4) Hybrid technology, which includes the fusion of natural EVs with synthetic liposomes, combining natural bio-targeting with tunable cargo capacity.

In addition to these four primary engineering approaches for EVs, modifying the culture conditions of EV-producing cells can further expand their therapeutic potential. Hypoxic preconditioning involves culturing cells under low-oxygen conditions before EV isolation. One study using DPSC-Exosomes showed upregulation of Hypoxia-inducible Factor 1-alpha (HIF-1α), VEGFA, and Kinase Insert Domain Receptor mRNA, as well as downregulation of Bone Morphogenetic Protein-2, resulting in EVs enriched with pro-angiogenic and pro-survival factors (Dou et al., 2018). Likewise, LPS-treated MSCs were found to activate AKT1/2 and skew macrophage polarization via NF-κB inhibition. This produced exosomes that suppressed TNF-α, IL-6, and IL-1 secretion to foster an anti-inflammatory environment (Li et al., 2022a). Treatment of BMMSCs with IFN-γ and TNF-α has also been shown to enhance the anti-inflammatory properties of derived exosomes by promoting the secretion of anti-inflammatory cytokines (Xu et al., 2019b). Beyond cytokine priming approaches, direct engineering of EV cargo provides another strategy to further enhance their therapeutic potential. For example, EVs transfected with miR-21–5p can enhance the expression of VEGF and HIF-1α in recipient cells by inhibiting Sprouty Homolog 2 and activating the PI3K/AKT and ERK1/2 signaling pathways, ultimately leading to increased cell proliferation. Various approaches and techniques for EV engineering have been summarized in Table 4.

Innovations in high-resolution imaging, microfluidic isolation, and nanoscale characterization have enabled precise identification and tracking of EVs from specific cell types. Omics-based approaches, including proteomics and RNA sequencing, now allow detailed profiling of EV cargo, revealing key molecular signals that modulate nerve regeneration and immune responses. Furthermore, bioengineering strategies are enhancing EV delivery and targeting, offering promising therapeutic avenues for improving functional recovery after PNI. As such, the application of EVs as natural carriers for various drugs extends beyond targeting specific diseases or delivery sites in the body. EV technology is now affianced to ameliorate the solubility issues of hydrophobic drugs and stability. By converting unstable drugs into natural amphipathic nanostructures within EVs, their encapsulation efficiency can be significantly increased, transforming them into more effective therapeutic agents (Tran et al., 2019).

Although engineering strategies often enhance EV potency, their impact on EV stability remains insufficiently characterized. Emerging evidence suggests that certain modifications, such as surface conjugation, cargo overloading, or nanomaterial incorporation, may alter membrane integrity, circulation half-life, and degradation susceptibility (Wang et al., 2025a). In some cases, engineered EVs may exhibit reduced stability compared to native EVs, leading to faster clearance or loss of bioactivity over time (Sanaee et al., 2023). However, systematic, side-by-side comparisons of stability between engineered and natural EVs are still lacking, representing a critical knowledge gap for clinical translation (Görgens et al., 2022). This raises an important practical concern: increased bioactivity must be balanced against the need for sufficient structural stability and functional persistence, especially for applications requiring storage, transport, or sustained release *in vivo*.

## 7. Clinical trials and EVs

While the preclinical evidence for the efficacy of EVs in promoting peripheral nerve regeneration is robust, their clinical translation for PNI remains in its early stages. Of the stem cell EV clinical trials found on (https://clinicaltrials.gov/ct2/home), none specifically target traumatic PNI currently. In broader PNI contexts, a recruiting trial (NCT05152368) is evaluating UCMSC-EVs for trigeminal neuralgia at a dose of 400 billion EVs per nasal puff, but no data has been formally published to date. No additional clinical trials investigating EV-based therapies for PNI were identified. However, meta-analyses of published clinical trials on EV-based therapy show a low incidence of adverse effects (4.4%, 95% CI: 0.7 – 22.2%) and are generally safe. In subgroup analyses, engineered EVs showed a significantly higher frequency of adverse events compared to non-engineered EVs (46.8 % vs. 1.5%, p = 0.003) (Van Delen et al., 2024). These adverse effects can be overcome by standardization of EV isolation and characterization data protocols. Nevertheless, potential risks, such as pathogen transmission, immune reactions, contamination, and off-target biodistribution underscore the importance of stringent quality control and standardized manufacturing processes to ensure the safety and efficacy of EV-based therapies (Terai et al., 2025).

Examining EVs in tangential clinical applications, such as with CNS diseases, can offer significant parallels for the prospects of EVs in peripheral nerve applications. A landmark trial (NCT07105371) on 18 patients with either Amyotrophic Lateral Sclerosis, Lewy Body Dementia, Kennedy Disease, or Congenital Myasthenic Syndrome was published recently using intranasal ADMSC-Exosomes. Remarkably, there were clinical improvements in 17 of 18 patients, and none of them experienced complications or unexpected deterioration (Prodromos et al., 2025). In other forms of CNS injury, there is indirect clinical evidence with autologous MSCs through the STARTING-2 trial (Stem Cell Application Research and Trials in Neurology-2) (NCT01716481) (Lee et al., 2022) with 39 chronic major stroke patients. EV particle count was directly associated with motor function improvement (p = 0.034), representing the first clinical trial to correlate circulating EVs with neuroregenerative plasticity in ischemic contexts (Bang et al., 2022).

With a minimal rate of adverse effects, and promising strides towards clinically significant improvements, EV-based therapies mark a transformative shift for neurotherapeutics. Importantly, these trials establish the needed proof-of-concepts to lay the foundation for future studies in PNI to verify whether similar EV-mediated neuroregenerative mechanisms can be harnessed to achieve functional restoration. A summary of published CNS-related EV clinical trials representing closely related diseases including preprints and trials with pending approval to date is provided in Table 5.

## 8. Perspective

Integration of EVs with biomaterials represents a promising strategy to overcome key limitations associated with EV delivery. Incorporation into nerve conduits, hydrogels, scaffolds, or injectable matrices can enhance EV stability, protect cargo integrity, and enable sustained, localized release at the injury site. Such approaches may improve therapeutic retention and bioavailability, which are critical for effective nerve regeneration. In parallel, engineering strategies such as modifying parent cells to overexpress specific miRNAs or proteins offer the potential to tailor EV cargo for targeted regenerative outcomes. However, the functional benefits of these approaches remain context-dependent, and systematic comparisons across delivery platforms are still lacking.

Although EVs have been widely investigated for their role in nerve repair, their therapeutic profile should be interpreted with caution. Stem cell–derived EVs can recapitulate several regenerative effects of their parent cells, including modulation of inflammation, promotion of axonal growth, and support of SC function, while avoiding risks associated with cell transplantation such as uncontrolled differentiation or immune rejection. Nevertheless, EV-based therapies do not fully replicate the dynamic and adaptive responses of living cells, and their efficacy may be limited by factors such as cargo variability, dosing constraints, and transient bioactivity. Preclinical studies have demonstrated encouraging outcomes using EVs delivered via direct injection or in combination with nerve conduits, with improvements in structural and functional recovery. However, key translational challenges remain unresolved, including optimal dosing strategies, long-term safety, biodistribution, and large-scale manufacturing consistency. Furthermore, the lack of standardized protocols complicates cross-study comparisons and hinders clinical progression.

A major barrier to clinical translation is not the lack of efficacy, but the absence of a clinically standardized, FDA-approved EV product with consistent potency. EV dosing remains poorly defined, with significant batch-to-batch variability driven by donor differences, culture conditions, and isolation methods. In addition, there is no universally accepted potency assay linking EV activity to functional nerve repair outcomes (Qiu et al., 2025). Storage further complicates translation, as EVs can lose bioactivity during freeze–thaw cycles or prolonged preservation. However, even optimal storage would not resolve the core issue of reproducibility. Consequently, most EVs remain at the research-grade level, lacking validated manufacturing and release criteria, limiting their reliability and preventing routine clinical use. Overall, EV-based therapies should be viewed as a complementary and evolving approach rather than a definitive replacement for stem cell therapies. Future advances will require integrated strategies that combine EV engineering, biomaterial-based delivery systems, and rigorous preclinical-to-clinical validation. Addressing these challenges will be essential to fully realize the potential of EVs for improving functional recovery in peripheral nerve injury.

## 9. Conclusions

EVs represent a promising class of cell-free therapeutic agents capable of delivering bioactive cargo, including miRNAs, proteins, and neurotrophic factors to target cells, thereby promoting neuroprotection, axonal regeneration, and modulation of neuroinflammation without the need for cell engraftment. Their ability to cross biological barriers, such as the blood–nerve and blood–brain barriers, and their potential for engineering-based targeting strategies highlight their translational appeal. However, it is premature to consider EVs as a superior alternative to stem cell–based therapies. Despite encouraging preclinical data and emerging clinical evidence in other disease contexts, several critical limitations remain insufficiently addressed. These include potential off-target effects, variability in cargo composition, and the risk of unintended immune modulation or pro-tumorigenic signaling, depending on EV source and context. In addition, long-term safety data is currently limited, and the biodistribution, persistence, and clearance of EVs *in vivo* remain incompletely understood.

From a translational perspective, manufacturing heterogeneity, lack of standardized isolation and characterization protocols, and batch-to-batch variability represent major barriers to clinical implementation. These challenges complicate reproducibility and hinder the ability to establish clear dose–response relationships and safety profiles. Future progress will depend on addressing these limitations through rigorous standardization, long-term *in vivo* safety studies, and well-designed clinical trials specifically in PNI models. Integrative strategies such as combining EVs with biomaterials–nerve conduits, hydrogels, and scaffolds–or engineering parent cells to optimize cargo composition may further enhance therapeutic precision and efficacy. Taken together, EVs serve as a promising platform with strong potential in supporting complex repair processes at the molecular level through their unique capacity for mechanistic specificity, favorable safety profile, and adaptable translational potential. As these technologies continue to improve, EV-based therapies may redefine the treatment landscape for PNI and provide long-term functional restoration for these patients.

## Figures and Tables

**Table 1:** Classification of EVs based on the size, shape, and genetic content.

EVs	Size (nm)	Shape	Formation	Genetic Content	References
Exosomes	30–150	Spheroid	Late endosome	mRNA, miRNA, non-coding RNAs lipids, proteins	Hánělová et al. (2024)
Microvesicles	100–1000	Irregular	Plasma membrane	mRNA, miRNA, non-coding RNAs cytoplasmic proteins membrane proteins	Clancy et al. (2023)
Apoptotic bodies	1000–5000	Irregular	Apoptotic cell membrane	nuclear fractions cell organelles	Kakarla et al. (2020)

**Table 2:** Summary of EV studies isolated from sources, indicating the regenerative potential of EVs and the underlying markers and pathways involved.

Study Description	EV Source	Particle Size (nm)	Dose	Key Research Findings	Markers And Pathways Involved	References
*In vitro* OGD hypoxic injury	SCs	140.5 – 148.9	1.6 ± 0.2 × 10^11^ particles/mL	↑ Neurite outgrowth***, **↑ Neuronal survival** & axonal area at 1*, 3**, 5 d** ↑ Differentiation and neurite outgrowth***	**↑ miR-21–5p**, PMP22, VEGFA, NGFR, S100β **↓ PTEN, ↑ PI3K/Akt pathway**	Cong et al. (2021)
*In vitro* SC/DRG co-culture & Rat Sciatic nerve 10 mm defect injury	ADMSCs	125.4 ± 0.1	20 μg/mL vs. 10 μg/mL	**↑ SC proliferation**, migration, & secretion of neurotrophic factors at 4*, 8 wks* ↑ DRG neurite length**, axonal myelination* & Repaired denervated muscle atrophy*	CD9^+^, CD63^+^ **↑ NF-H**	Chen et al. (2019)
Mice spared nerve injury	Placental mesenchymal stem cell-derived	100–200	0.5 μg/μL	**↑ 50% paw withdraw threshold** from 8 to 35 d**, ↓ Inflammatory factors** ↑ Anti-neuroinflammatory activity until 35 d	**↑ miR-26a-5p ↓ Wnt5a** Regulation of Wnt5a/Ryk/CaMKII/NFAT pathway	Lu et al. (2022)
Mice DPN	MSCs	>100	1 × 10^9^ particles/injection	**↑ NCV** at 2*, 4 wks**, **↓ Mechanical & thermal stimuli threshold** at 2*, 3*, 4 wks* ↓ Endothelial cell & inflammatory monocyte activation in the peripheral blood at 2*, 4 wks** ↑ Regional blood flow by 81%	**↑ TNFα, IL-1β ↓ TLR-4****/NF-****κ****B** pathway	Fan et al. (2021)
Mice Sciatic nerve crush injury	dMSCs	194 ± 50	dExo (1 × 10^10^ particles) in 50 μL pre-gel solution (10 mg/mL)	**↑ DRG neurite outgrowth** at 7 d ↑ Foot pressure*, perpendicular AEP**, PEP*, stance duration**, & swing duration* ↑ Sensorimotor recovery* **↑ NCV** at 14 **, 21d*, ↑ Remyelination at 21 d**	Alix^+^, CD9^+^ **↓ CSF-1/CSF-1R** pathway	Liu et al. (2024)
Mice Sciatic nerve crush injury	GMSCs	103.8 ± 2.1	40 μg in 20 μL	↑ Axon regeneration*, ↑ SFI** & Toe spread score** at 1 m	CD63^+^, CD9^+^ **↓ c-Jun ↑ EGR2/KROX20**, GFAP	Mao et al. (2019)
Rat Sciatic nerve crush injury	ADMSCs	86.07	50 μg/mL 1 μg/μL	↓ Number of autophagosomes***, autophagy-related protein expression** **↑ Myelin sheath regeneration** at 8 wks**	**↓ miR-26b ↓ Kpna2** in injured SCs	Yin et al. (2021)
Rat Sciatic nerve crush injury	BMMSCs	118.3	4 μL exosomes	**↑ Regenerated axons**, diameter, SFI index (0.34 ± 0.07 vs. −0.76 ± 0.09)**, LTP **, NCV*, & thermal latency scores (23.61 ± 5.04s vs. 58.6 ± 11.12s)**	CD63^+^, CD44^+^, CD9^+^ **↑ VEGFA, S100β, NGFR, PMP22**	Zhao et al. (2020)
Rat Sciatic nerve crush injury	BMMSCs	40–300	100 μL exosomes	**↑ SFI** at 2*, 4*, 6 wks*, **↑ Myelin & axon regeneration** at 4 wks*	**↑ GAP-43**	Ma et al. (2017)
Rat Sciatic nerve crush injury	MSCs	113 ± 32	(1.75 ± 0.7) × 10^11^ particles/mL, 3 μL in 50 μl collagen solution/Rat	**↓ Muscle atrophy (19%) ↑ Muscle fiber diameter (33.3%)** at 30 d ↓ Apoptosis (50%)*	CD9^+^, CD81^+^, TSG101^+^ **↑ NF-200, GAP-43**	Demyanenko et al. (2022)
Rat Sciatic nerve crush injury	PRP-MSCs	102.1 ± 58.7	2.7 × 10^11^ particles/mL; 10, 50, & 100 μg/mL	↑ SFI** & CMAP**, ↓ Latency* ↑ Neural repair at 28 d**	CD9^+^, CD63^+^, CD81^+^ **↑ GDNF ↑ PI3K/Akt** pathway	Zhang et al. (2024)
Rat Sciatic nerve crush injury	ADMSCs	N/A	N/A	**↑ SC proliferation (Ki67**^**+**^ **expression 31.92 ± 5.08%), axonal regeneration**, neurite length (55.3 ± 28.7 μm) **SFI (32.20 ± 23.88 vs. 38.98 ± 5.36)**	CD63^+^, CD9^+^ **↑ S100, NF**	Bucan et al. (2019)
Rat Sciatic nerve crush injury	DPSCs	N/A	N/A	↓ Autophagy of SCs* ↑ Functional recovery*	**↑ miR-122–5p ↓ P53** expression, via miR-122–5p/P53 pathway	Chai et al. (2024)
Rat Sciatic nerve crush injury	ECs	128.8	1 μg	**↓ Apoptosis rate** of SCs (from 4.01% to 2.07%), ↑ Functional recovery** at 14 d, ↑ SFI at 14**, 28 d** ↑ Gastrocnemius muscle weight****, mean fiber diameter* & **axonal growth** in vitro**	CD9^+^, CD81^+^, TSG101^+^ **↑ miR199–5p** PI3K/AKT/PTEN pathway	Huang et al. (2023)
Rat Sciatic nerve 5 mm defect injury	hUCMSCs	80 – 650	1 μg/μL	↑ SFI*, Regenerated nerve diameter**, number of myelinated fibers**, & gastrocnemius muscle recovery* at 4 wks	CD3^+^ **↑ NF-200, Cy3, S100, MBP, IL-10 ↓ IL-6, IL-1β**	Ma et al. (2019b)
Rat Sciatic nerve defect 5 mm injury	OECs	100.1 ± 26.6	10^8^ particles/mL, as EV + Matrigel and EV + conduit	**↑ Neurite outgrowth (234.7 ± 41.4 μm)** compared to **control (199.3 ± 37.3 μm)** *in vitro*, **↑ Number of axons** in EV group (7830 ± 377) compared to control (2818 ± 321) at 8 wks post-surgery, ↑ SFI**, Myelination, and axonal regeneration*	Alix^+^, TSG101^+^, CD63^+^, Calnexin **↑ NF160**	Xia et al. (2019)
Rat Sciatic nerve 5 mm defect injury	Human embryonic stem cells	N/A	45 μg MV filled as silicone tubes	**↑ Functional recovery** at 12 wks*	**↑ Nestin, Pax6, Beta III Tubulin, Sox 1, Sox 2**	Chen et al. (2020)
Rat Sciatic nerve 10 mm defect injury	ADMSCs	30 – 150	1 μg/μL	**↑ Nerve regeneration** & gastrocnemius muscle recovery at 8 wks*	CD9^+^, TSG101^+^ Restoration of **NT-3, NF200, S100**	Yang et al. (2021)
Rat Sciatic nerve 10 mm defect injury	GMSCs	102	10 μg exosomes/10 μL PBS	↑ SC proliferation*, DRG neurite outgrowth*, Myelination*, ↑ Nerve & muscle function**	CD9^+^, CD63^+^ **↑ NF200, S100**	Rao et al. (2019)
Rat Sciatic nerve 12 mm defect injury	OECs and UCMSCs	75.28	400 μg/mL exosomes	**↑ Number of OECs** under hypoxic conditions **(4.62-fold)** compared to normal (1.76-fold)**, ↑ Number of migrating OECs (29.6 ± 3.86) & **Sensorimotor function** at 4*, 8 wks**	HSP70^+^, CD9^+^, CD63^+^ **↑ GFAP, BDNF, NF200, S100**	Zhang et al. (2020)
Rat DPN	BMMSCs and NGCs	211.8 ± 76.5	20 μg/mL exosomes	↑ NCV****, **↑ CMAP** at 4***, 6***, 8 wks**** Improved sciatic nerve morphology at 60 d*** after crush & 90 d* after transection	CD9^+^, CD81^+^	Singh et al. (2021b)
Rat Sciatic nerve CCI	MSCs	40 – 110	10 μL (1.2 mg/mL) exosomes	**↑ Mechanical allodynia & thermal hyperalgesia** at 7***, 14***, 21 d***	CD63^+^, TSG101^+^ **↓ miR-181c, IL-6, IL-1β, TNF-α, COX-2**	Zhang et al. (2022)

Showed ↑ = increase or upregulated, ↓ = decrease or downregulated, d = days, wks = weeks, m = months, significance as * = p < 0.05, ** = p < 0.01, *** = p < 0.001 & **** = p < 0.0001 compared to control.

Abbreviations: OGD: Oxygen glucose deprivation; OEC: Olfactory ensheathing cell; LTP: Latency of thermal pain; SFI: Sciatic function index; KPNA2: Karyopherin Subunit Alpha 2; NCV: Nerve conduction velocity; CMAP: Compound muscle action potential; LPS: Lipopolysaccharide; NGC: Nerve guidance conduit; DPN: Diabetic peripheral neuropathy; CCI: Chronic constriction injury; AEP: Anterior Extreme Position; PEP: Posterior Extreme Position.

**Table 3:** Summary of stem cell-derived EVs as delivery molecules (nanocarriers) in PNI studies.

Therapeutic Molecule	EV Source	Proposed Application in Disease	Finding And Outcome	References
miR-21	SCs	DPN *in vitro*	**↑ Neurite growth induction (28.36 ± 6.629 μm)** in high glucose conditions**	Liu et al. (2022c)
siRNA targeting Ago2	BMMSCs	Rat Sciatic nerve crush injury	↑ Regeneration of peripheral nerves**, ↑ **SFI (−0.34 ± 0.07)** at 28d**, **↑ Myelinated nerve fibers (8100 ± 660)** & myelin fiber diameter (1.86 ± 0.23 μm) ↑ VEGFA*	Zhao et al. (2020)
NT-3	ADMSCs	Rat Sciatic nerve defect model	Sustained release (80%) of EVs with NT-3, ↑ S100* & NF200*, **↑ Nerve regeneration, SFI, CMAP** & gastrocnemius muscle ratio at 2 wks*	Yang et al. (2021)
miR-146a	MSCs	Rat DPN	**↑ Mechanical & thermal** stimulus thresholds, **↓ NCV** at 4** wks, **↑** Functional recovery at 2*, 4 wks**	Fan et al. (2021)
PpyNPs-loaded liposomes	BMMSCs and NGCs	Rat DPN	**↑ NCV & CMAP** in DPN rats at 4 wks*	Singh et al. (2021b)

Showed ↑ = increase or upregulated, ↓ = decrease or downregulated, d = days, wks = weeks, m = months, significance as * = p < 0.05, ** = p < 0.01, and *** = p < 0.001 compared to control.

Abbreviations: NT-3: Neurotrophin-3; PpyNPs: Polypyrrole nanoparticles; Ago2: Argonaute; NGC: Nerve guidance conduit.

**Table 4:** Various bioengineering strategies to improve and promote properties of EVs.

Type of EVs Engineering	Mediators	Source	Outcome	References
Genetic pathways	Transfected with BMP2 gene	BMMSCs	↑ Therapeutic effects & accelerating effect in bone healing	Li et al. (2022b)
Biochemical pathways	LPS pre-conditioning	BMMSCs	↑ M2 macrophage polarization *in vitro* ↓ Inflammation	Xu et al. (2019b)
	miR-21–5p impression by Fe_3_O_4_, Iron oxide nanoparticles	BMMSCs	↑ Activation of PI3K/AKT & ERK1/2 signaling	Wu et al. (2020a)
	Fe_3_O_4_, Iron oxide nanoparticles	MSCs	↑ Anti-inflammatory response & angiogenesis, ↓ Apoptosis	Kim et al. (2020)
	miR-219a-2–3p, Insulin-like growth factor-1	NSCs	↑ Neuroprotection, ↓ NF-κB pathway & neuroinflammation	Ma et al. (2019a)
	BDNF	UCMSCs	↑ Nerve fiber regeneration, synaptic connections & myelin sheath	Liu et al. (2023)
	FK506	ADMSCs	↑ HDAC, APP, ITGB1, & nerve regeneration	Rau et al. (2021)
	FK506	ADMSCs	↑ HSPA8, EEF1A1, & nerve regeneration, ↓ Autophagy	Kuo et al. (2021)
	285-amino-acid-long Heme-Binding Protein 1	Pericyte	↑ Neurovascular regeneration, ↓ Vascular permeability	Yin et al. (2024)
Mechanical pathways	Bioreactors	UCMSCs	↑ TGF-β1, Smad2/3, angiogenesis, & regeneration	Yan and Wu (2020)
	LIPUS	SCs	↑ PI3K-Akt-FoxO signaling enhances nerve regeneration	Ye et al. (2023)
Culture conditions	Low vascularization bioactivity	MSCs	↓ Cell seeding density resulted in increased production	Patel et al. (2017)
	Hypoxia	MSCs	↑ VEGF, angiogenesis, & functional recovery	Mu et al. (2022)
	Hypothermia	Microglial	↑ Neurite outgrowth & synapse recovery	Wang et al. (2023)

Abbreviations: LIPUS: Low-intensity pulsed ultrasound; HDACs: histone deacetylases; APP: amyloid-beta A4 protein; and ITGB1: integrin beta-1.

**Table 5:** Summary of Completed Clinical Trials for EVs as biomarkers or treatment for various CNS pathologies.

Study/Clinical Trial ID	Country/Location	Condition(s)	EV Type	Clinical Outcomes/Study Objectives	Adverse Effects	Mechanistic/Translational Insight	Takeaways for future PNI studies	References
NCT07105371	USA	ALS, KD, CMS, LBD	AlloEx ASC-Exosomes	↑ Strength (5 kg, 5 reps), ↓ Pain (Level 1–2 out of 10), ↓ Cognitive impairment	None reported	Demonstrates functional improvement in neurodegenerative conditions using MSC-EVs	Potential feasibility study of EV-based therapies in PNI	Prodromos et al. (2025)
NCT04388982	China	Alzheimer’s disease	Allogenic human ASMSC-EVs	↑ Cognitive function & hippocampal volume, ↓ Amyloid and tau deposition;	None reported	Suggests disease-modifying potential in neurodegeneration	Potential neuroprotective applications of EVs in PNI	Xie et al. (2023b)
NCT01716481	South Korea	Chronic ischemic stroke	Circulating EVs after MSC injection	↑ Motor function & motor network integrity; ↑ miRNA-18a-5p, Modulation of BDNF, VEGF, NGF	None reported	Links EV-associated miRNAs with neuroregeneration and plasticity	Potential EV-associated miRNAs changes in PNI	Bang et al. (2022)
NCT05279599	China	Traumatic brain injury	Circulating EVs	Correlation with disease progression and outcomes	None reported	Investigates EVs as acute injury biomarker in CNS trauma	Potential EV-based prognostic markers for PNI severity and progression	Pending re-approval https://clinicaltrials.gov/study/NCT05279599
ICSS-2019-019 NCT04202783	USA	Neuralgia	sEVs	Pain modulation via anti-inflammatory effects	None reported	Investigates the link between EVs and NF-κB–mediated pain pathways	Potential EV-based targets of inflammatory pain pathways in PNI	Pending re-approval https://clinicaltrials.gov/study/NCT04202783
ICSS-2018–018 NCT04202770	USA	Depression/neurodegenerative disease	sEVs	CNS targeting via ultrasound delivery	None reported	Investigates enhanced EV brain delivery using physical targeting methods	Potential feasibility of ultrasound-guided EV delivery in PNI therapies	Pending re-approval https://clinicaltrials.gov/study/NCT04202770
X121207003 NCT01860118	USA (Birmingham)	Parkinson’s disease	sEV proteomic EVs	Biomarker identification (LRRK2, protein changes)	None reported	Investigates proteomic insights into EV cargo in neurodegeneration	Potential EV proteomics in identifying regenerative biomarkers in PNI	Completed https://clinicaltrials.gov/study/NCT01860118

Abbreviations: EVs: extracellular vesicles; MSC: mesenchymal stem cell; ADMSC: adipose-derived mesenchymal stem cell; sEV: small extracellular vesicles (<200 nm; “exosomes” in the original clinical trial; the term should be used with caution per MISEV 2023); PNI: peripheral nerve injury; USA: United States of America; ALS: Amyotrophic Lateral Sclerosis; KD: Kennedy Disease; CMS: Congenital Myasthenic Syndrome; LBD: Lewy Body Dementia; and LRRK2: Leucine-rich repeat kinase 2.

## Abbreviations

ADMSCs: Adipose derived stem cells
Bax: Bcl-2-associated X protein
Bcl-2: B-cell lymphoma 2
BDNF: Brain derived nerve factor
BMMSCs: Bone marrow mesenchymal stem cells
βFGF: Beta fibroblast growth factor
CAMKK1: Calcium/Calmodulin-Dependent Protein Kinase 1
COS: Chitosan oligosaccharides
COX: 2 Cyclooxygenase-2
CMAP: Compound muscle action potential
DPSCs: Dental pulp stem cells
DPN: Diabetic peripheral neuropathy
DRGs: Dorsal root ganglion
EGR2: Early Growth Response 2
ERK: Extracellular signal-regulated kinase
p-ERK: phosphorylated extracellular signal-regulated kinase
EGF: Epidermal growth factor
EGFR: Epidermal growth factor-receptor
EVs: Extracellular vesicles
ECH-EVs: Electroconductive EVs
FOXO: Forkhead box O
GAP43: Growth associated protein 43
GFAP: Glial Fibrillary Acidic Protein
GDNF: Glial Cell Line-derived Neurotrophic Factor
HO-1: Heme oxygenase-1
HAMA: Hyaluronic acid methacrylate
HIF-1α: Hypoxia-inducible factor 1-alpha
IM: Intramuscular IV Intravenous
IL-1β: Interleukin-1 beta
iPSC: Induced pluripotent stem cells
IFN-γ: Interferon-gamma
iNOS: Nitric oxide synthase
LPS: Lipopolysaccharide
LIPUS: Low-intensity pulsed ultrasound
MAPK: Mitogen-activated protein kinase
MEK: MAPK/ERK kinase
mTOR: Mammalian target of rapamycin
MSCs: Mesenchymal stem cells
NCV: Nerve conduction velocity
NF200: Neurofilament 200
NF-kB: Nuclear Factor kappa-light-chain-enhancer of activated B cells
NGF: Nerve growth factor
NGFR: Nerve Growth Factor Receptor
NLRP3: Pyrin domain-containing 3
Nrf2: Nuclear factor erythroid 2–related factor 2
OGD: Oxygen-glucose-deprivation
OECs: Olfactory ensheathing cells
PC12: Rats adrenal pheochromocytoma cells
PGE2: Prostaglandin E2
PNI: Peripheral nerve injury
PpyNPs: Polypyrrole nanoparticles
PNS: Peripheral nervous system
PTEN: Phosphatase and tensin homolog
PI3K: Phosphoinositide 3-kinase
ROS: Reactive oxygen species
SCs: Schwann cells
SH-SY5Y: Human neuroblastoma cell line
SFI: Sciatic Functional Index
SKP: Skin precursor cells
TGF-β1: Transforming Growth Factor Beta 1
TSG-6: TNF-stimulated gene-6
TLR4: Toll-like receptor 4
TNF-α: Tumor Necrosis Factor-alpha
TRAF6: TNF receptor-associated factor 6
TSC: Tuberous sclerosis complex
VEGFA: Vascular endothelial growth factor A
UCMSCs: Umbilical cord stem cells
WJ-UCMSCs: Wharton’s Jelly-derived Umbilical cord mesenchymal stem cells
